# Protein glycoxidation in neuropsychiatric disorders—from basic research to clinical practice

**DOI:** 10.1016/j.redox.2026.104190

**Published:** 2026-04-27

**Authors:** Wiktor Orlof, Mateusz Maciejczyk

**Affiliations:** a Department of Psychiatry, The Faculty of Medicine, Medical University of Bialystok, 2 Wolodyjowskiego Street, Bialystok, 15-272, Poland; b Department of Hygiene, Epidemiology and Ergonomics, Medical University of Bialystok, 2c Mickiewicza Street, Bialystok, 15-022, Poland

**Keywords:** Glycation, Advanced glycation end products (AGEs), Receptor for advanced glycation end products (RAGE), Neuropsychiatric disorders

## Abstract

This review integrates current findings on protein glycation, glycoxidation, and carbonyl stress in selected neurodegenerative and neuropsychiatric disorders, with a particular focus on mechanistic pathways relevant to Alzheimer's disease (AD), Parkinson's disease (PD), schizophrenia, and depression, as well as on their potential clinical relevance, including biomarker development and antiglycation interventions. These processes are increasingly recognised as cross-cutting features in various neuropsychiatric conditions. Despite this mechanistic relevance, no neuropsychiatric drugs have been convincingly demonstrated to exert direct antiglycation activity *in vivo*. The majority of published findings concern indirect modulation of carbonyl stress, redox imbalance, and advanced glycation end products (AGEs) and their receptor (RAGE) signalling rather than direct inhibition of glycation. AGEs, particularly the lysine-derived adducts Nε-(carboxyethyl)lysine (CEL) and Nε-(carboxymethyl)lysine (CML), show potential as diagnostic and prognostic biomarkers in neurodegenerative diseases, although further clinical validation is required. Modulation of protein glycation, carbonyl stress, and AGE–RAGE signalling has emerged as a common mechanistic denominator in various therapeutic strategies explored in neuropsychiatric disorders.

## 1 Introduction

Neuropsychiatric conditions such as Alzheimer's (AD) and Parkinson's (PD) disease predominantly emerge in later life and are characterised by a gradual erosion of neuronal integrity, which ultimately manifests as a progressive dysfunction of the central nervous system networks rather than as an isolated loss of individual neurons. Epidemiological data suggest a substantial and progressive increase in the demographic burden of these disorders, with estimates indicating an approximate doubling every two decades [1,2]. According to this forecast, the global number of patients is expected to reach around 80 million by 2040 [2]. In individuals older than 65 years, AD accounts for a majority of dementia diagnoses, with reported proportions typically ranging between approximately 60% and 65% [3]. Against this epidemiological backdrop, AD and PD exhibit distinct clinical phenotypes. AD causes a gradual deterioration of memory, cognitive functions, and orientation [4]. In contrast, PD is characterised by a parkinsonian syndrome marked by tremor, muscular rigidity, and prominent non-motor manifestations [5].

Psychiatric disorders, including depression and schizophrenia, are prevalent across the lifespan. Depression constitutes a substantial component of the global burden of psychiatric diseases, affecting heterogeneous populations throughout life [6]. It presents as prolonged low mood, lack of interest, psychomotor retardation, and cognitive impairment [7]. According to the World Health Organization (WHO), ∼3.8% of the world's population, or about 280 million people, is living with depression. Depression is projected to be one of the leading causes of disease worldwide by 2030 [8]. Schizophrenia is the most common chronic psychotic disorder with a stable, low-prevalence distribution across populations (∼1%) [9]. Symptoms of schizophrenia include hallucinations, delusions, disorganised thinking, and/or mood disorders [9,10].

Effective treatment and prevention of neurodegenerative disorders require a thorough understanding of their underlying biological mechanisms. However, neuropsychiatric disorders appear to arise from convergent and incompletely characterised biological processes. Current neurodegeneration models emphasise the coexistence of pathogenic protein accumulation and chronic inflammatory responses as interrelated features of disease progression [11]. Indeed, AD is characterised by the accumulation of extracellular amyloid beta (Aβ) plaques and intracellular tau protein tangles [12], while PD involves the accumulation of alpha-synuclein (αSyn) as Lewy bodies [13]. The pathogenesis of schizophrenia and affective disorders is associated with altered neurotransmission and chronic inflammation [14].

In this context, advanced glycation end products (AGEs) are increasingly considered within the mechanistic framework of neuropsychiatric disorders. They are formed during a multi-stage, non-enzymatic glycation process involving reducing sugars and protein-associated amino groups [15]. In the final stage, AGEs form through condensation, coupling, polymerization, hydrolysis, or decomposition reactions. AGEs were first described over 100 years ago. However, the structural–functional relationships governing AGE-mediated cellular effects remain incompletely resolved [16]. Previous studies indicate that AGEs accelerate Aβ and phosphorylated tau (p-tau) aggregation in individuals with AD, contributing to the formation of disease-specific protein aggregates [1]. Carbonyl stress may exacerbate inflammatory and oxidative conditions, thereby compromising neuronal integrity over time [5,12,13]. Indeed, AGEs interact with the receptor for advanced glycation end products (RAGE), which is expressed primarily on the surfaces of immune and nervous system cells [17,18]. RAGE engagement initiates a cellular response characterised by redox imbalance and altered inflammatory signalling. This response is associated with increased generation of reactive oxygen (ROS) and nitrogen species (RNS), as well as upregulation of vascular cell adhesion molecule-1 (VCAM-1) and intercellular adhesion molecule-1 (ICAM-1) [19]. Furthermore, activation of several signalling pathways, including nuclear factor kappa B (NF-κB), has been demonstrated to occur as a consequence of AGE–RAGE interactions, resulting in elevated production of pro-inflammatory cytokines such as tumour necrosis factor-α (TNF-α), interleukin-6 (IL-6), interleukin-1β (IL-1β), interferon-γ (IFN-γ), and monocyte chemoattractant protein-1 (MCP-1) [20]. Understanding the mechanisms underlying glycation and the effects of AGEs on cells and tissues may facilitate the identification of effective RAGE inhibitors. Such inhibitors may represent a useful strategy for reducing carbonyl stress [21,22].

This review synthesises current evidence on protein glycation, glycoxidation, and carbonyl stress in the pathogenesis of neuropsychiatric disorders. These processes are increasingly recognised as key contributors to both disease onset and progression in neurodegenerative disorders (AD and PD) and mental disorders, including schizophrenia and depression. Attention is drawn to compounds with reported antiglycation or glycation-modulating properties. The search for new therapeutic and diagnostic methods is particularly relevant given the scale of the problem posed by neurodegenerative and mental disorders.

## 2 Glycation mechanism

The first mention of glycation in the literature dates back to 1912, when the French scientist Camille Maillard observed and described it [15]. His experiment involved heating amino acids with reducing sugars (containing a free aldehyde (-CHO) or ketone (-C=O) group), resulting in the formation of a brown product. Accordingly, protein glycation is commonly described as a “browning reaction”, during which reducing sugars form covalent bonds with free amino groups of proteins, including the N-terminal residues of lysine (Lys) or arginine (Arg) [23]. Individual sugars differ in their ability to bind to amino groups. Monosaccharides react faster than disaccharides, and glucose-6-phosphate (G6P) undergoes glycation more readily than glucose [24,25]. Similarly, fructose consumed by individuals with diabetes as a sugar substitute is more reactive than glucose [26,27]. In the initial stage of glycation, an unstable Schiff base, N-substituted glucosamine, is formed, which undergoes intramolecular rearrangement and decomposes into smaller molecules. These compounds contain a reactive O=C–C–N grouping and are referred to as Amadori products (APs) [28,29]. Schiff base intermediates, together with APs, represent the initial adducts formed during the early phase of protein glycation [5]. In the next stage, carbon and nitrogen atoms from carbonyl groups can undergo coupling to form heterocyclic compounds, including pyrazines. These compounds contribute to the characteristic products of the Maillard reaction, including flavour and aroma compounds [30]. Multiple pathways underlying the formation of AGEs operate within the Maillard reaction. Among them, the Wolff pathway is characterised by the autoxidation of glucose to arabinose, followed by its reaction with protein-bound amino groups of Lys or Arg [31]. More reactive Schiff base derivatives may also form *in vivo*, which undergo reverse aldol reactions followed by autoxidative decomposition, leading to the generation of reactive α-dicarbonyl species—namely methylglyoxal (MGO) and 3-deoxyglucosone (3-DG) produced in the Namiki pathway [31]. In contrast, in the Hodge pathway, APs can undergo non-oxidative rearrangement and deamination, which also leads to the formation of α-dicarbonyl precursors of AGEs [31]. These compounds include 3-DG (the predominant product) and MGO, formed by the autoxidation of glucose to glyoxal (GO). In addition, α-dicarbonyls can also be formed by fragmentation of glycerol-3-phosphate (3 PG) in the non-oxidative rearrangement of Amadori adducts with fructose-3-phosphate (F3P) in the polyol pathway. An alternative glucose-processing mechanism, known as the polyol pathway, involves the reduction of glucose to sorbitol by aldose reductase (AR), after which it is further metabolised to fructose by sorbitol dehydrogenase (SDH). Under hyperglycaemic conditions, this pathway is over-activated [32,33]. This mechanism promotes osmotic stress, resulting in cellular injury and contributing to complications such as neuropathy and diabetic retinopathy [34,35]. In addition, excessive consumption of redox coenzymes (reduced nicotinamide adenine dinucleotide phosphate (NADPH) and oxidised nicotinamide adenine dinucleotide (NAD^+^)) in the sorbitol pathway also has other consequences. Consequently, these cofactors are less available for other essential cellular processes, such as glutathione (GSH) regeneration [32,33]. Insufficient cellular reductase activity (e.g., glyoxalases 1 and 2 (GLO1 and GLO2), aldehyde dehydrogenase (ALDH), AR, and glyoxylate reductase (GLYR)), or an excess of substrates (e.g., glucose in diabetes), leads to the accumulation of reactive carbonyl species (RCS), including GO, MGO, and 3-DG [36]. This increases the risk of intracellular damage by altering protein function and structure, as well as inducing oxidative stress [32,37].

In addition to APs, GO and MGO also undergo further transformations in cells and tissues (oxidation, hydrolysis, polymerization, or polycondensation) to form AGEs. Glycation can extend beyond proteins and involve lipids and nucleic acids [22,38]. If the process involves deoxyribonucleic acid (DNA), reactive aldehyde species can bind to nitrogenous bases (adenine and guanine), which can lead to mutations, as well as replication and transcription defects [39].

Glycation is a multi-step process whose rate depends on the concentration of substrates and the duration of contact between them [15]. In contrast, AGEs accumulation is modulated by the capacity of repair mechanisms and excretory pathways [17,38,40]. Under homeostatic conditions, AGEs are degraded within lysosomal compartments [41]. The degradation products of glycated proteins are secreted outside the cell. These degradation products are typically released into the extracellular space and subsequently enter the circulation, where the kidneys excrete them with urine. A certain amount of AGEs can also be removed from the circulation by other means, including the endothelium and hepatocytes [41,42].

**Fig. 1:**
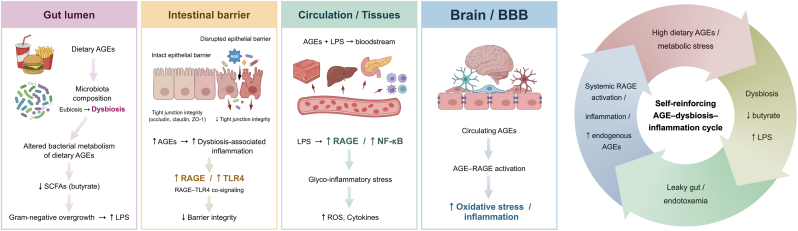
**Gut microbiota–advanced glycation end products (AGEs)–receptor for advanced glycation end products (RAGE) axis and its systemic consequences.** Dietary AGEs enter the gut lumen and interact with the intestinal microbiota. Alterations in microbiota composition—particularly a shift from eubiosis to dysbiosis—influence the microbial metabolism of dietary AGEs. Dysbiosis is often characterised by reduced abundance of butyrate-producing bacteria and overgrowth of Gram-negative taxa. This leads to decreased production of short-chain fatty acids (SCFAs), especially butyrate, and increased levels of lipopolysaccharide (LPS). At the intestinal barrier, reduced SCFA availability and elevated LPS levels disrupt epithelial integrity and cause tight junction dysfunction. Increased intestinal permeability allows the translocation of LPS and AGEs into the systemic circulation. In blood and peripheral tissues, LPS stimulates RAGE expression and activates nuclear factor kappa B (NF-κB) signalling pathways. These processes increase the production of reactive oxygen species (ROS) and pro-inflammatory cytokines. Circulating AGEs can interact with RAGE at the brain level and the blood–brain barrier (BBB), promoting oxidative stress and inflammatory responses within the neurovascular unit. This contributes to endothelial dysfunction and increased BBB vulnerability. High dietary AGEs, dysbiosis, impaired intestinal barrier, systemic inflammation, and RAGE activation form a self-reinforcing AGE–dysbiosis–inflammation cycle. This cycle amplifies oxidative stress and inflammatory signalling and can contribute to the pathophysiology of neuropsychiatric disorders.

It should be noted that some AGEs are also exogenous (e.g., derived from food) [15]. Foods such as baked bread (with a brown crust), grilled meat, and peanut butter are particularly rich in AGEs. These compounds are present in a range of processed foods and beverages, including fructose-rich carbonated drinks, alcoholic beverages (wine and beer), and other products high in simple sugars [15,43]. Despite this, the contribution of exogenous AGEs to the total body AGEs burden appears limited, as these are resistant to both enzymatic and chemical hydrolysis and are only absorbed to a limited extent from the gastrointestinal tract [15,44]. The gut microbiota indirectly modulates endogenous AGEs burden and AGE–RAGE signalling by influencing dietary AGEs metabolism, intestinal barrier integrity, and inflammatory tone; dysbiosis may increase translocation of AGEs and bacterial products such as lipopolysaccharide (LPS), thereby amplifying systemic AGE–RAGE activation, carbonyl stress, and inflammatory signalling [45,46]. Although dietary AGEs contribute modestly to total body AGEs load under normal renal function, they may still act as biologically relevant circulating ‘amplifiers’ of AGE–RAGE signalling—particularly in metabolic or inflammatory states and when gut barrier function is compromised [47]. Their bioavailability averages 10–30% of the amount ingested with food, and approximately 30% of AGEs absorbed via this route are excreted in the urine within two days [48]. This absorption does not imply proportional brain accumulation but may indirectly affect blood–brain barrier (BBB) function via systemic inflammatory and vascular stress. However, in patients with renal impairment, the excretion of AGEs in urine may decrease to as low as 5% [48,49]. Therefore, shifts in dietary habits that decrease exogenous AGEs intake may contribute to a reduced AGEs burden in the body, accompanied by diminished oxidative stress and inflammatory processes induced by AGEs [30,48]. Circulating AGEs affect the central nervous system (CNS) indirectly via the neurovascular unit, where activation of the AGE–RAGE axis in brain endothelial cells promotes oxidative stress, inflammation, and BBB dysfunction. Available evidence indicates that brain AGEs are generated predominantly endogenously, whereas dietary AGEs mainly act as circulating amplifiers of AGE–RAGE signalling, particularly in the context of metabolic disorders [50,51] (Fig. 1). These gut systemic mechanisms represent only one context in which AGE–RAGE signalling may become biologically relevant; more broadly, RAGE functions as a pleiotropic pattern-recognition receptor involved in inflammatory, reparative and stress-related signalling in multiple tissues.

## 3 Interaction between AGEs and RAGE

An association between increased protein glycation and organ damage has been observed [[52], [53], [54], [55]]. This damage primarily affects blood vessel walls and nerve cells [17,56]. AGEs damage cellular systems both directly and indirectly, primarily by amplifying carbonyl stress, oxidative stress, and lipoxidation-related injury [57,58]. Through the oxidation of unsaturated fatty acids, lipoxidation produces reactive aldehyde intermediates such as 4-hydroxynonenal (4-HNE) and MDA, which subsequently lead to advanced lipoxidation end products (ALEs) [58,59].

In this review, we use the term glycation to denote the non-enzymatic reaction of reducing sugars and RCS with amino groups of proteins and other biomolecules, leading to the formation of early glycation adducts and, ultimately, AGEs [22,60]. We use glycoxidation in a narrower mechanistic sense to describe the oxidative transformation of glycated intermediates and proteins, a process that enhances RCS generation and promotes the accumulation of AGEs and related oxidative modifications [61]. By contrast, carbonyl stress is used as a broader pathophysiological framework referring to the excessive accumulation of RCS and their downstream consequences, encompassing glycation, glycoxidation, and related carbonyl-driven damage such as lipoxidation [62,63]. This distinction is important because not all oxidative stress is glycoxidative in origin, whereas glycoxidation represents one mechanistic route through which carbonyl stress may amplify AGE–RAGE signalling, redox imbalance, and neuroinflammatory injury [[60], [61], [62]].

Among cell-surface receptors, RAGE has been identified as a key binding partner for AGEs and was first described following its isolation from human lung tissue in 1992 [64]. RAGE is expressed most abundantly in type II alveolar epithelial cells, in which they contribute critically to the maintenance of the alveolar–capillary barrier [65]. The presence of RAGE has also been confirmed on hepatocytes, phagocytes, endothelial cells, blood vessel muscles, and cells of the nervous system [53]. Ligand-RAGE signalling modulates cellular responses, ranging from proliferation and inflammation to autophagy and apoptosis, depending on the context and intensity of activation. Evidence suggests that RAGE contributes to tissue regeneration, particularly during muscle and nerve repair following injury. In skeletal muscle, RAGE expression appears to be dynamically regulated, and increased receptor levels have been associated with the promotion of myoblast differentiation [66]. Its activation is associated with recruitment of reparative cell populations, including stem and Schwann cells [67]. In addition, this signalling context has been implicated in neurogenic processes that support structural remodelling and functional recovery following neural injury [68]. However, in the adult hippocampus—where ongoing neurogenesis depends on tightly regulated neural stem/progenitor cell maintenance and niche homeostasis—experimental evidence indicates that sustained carbonyl stress and AGEs accumulation predominantly exert anti-neurogenic effects [69,70]. In models of metabolic disease, AGE-related mechanisms have been associated with reduced generation and/or survival of new neurons in the dentate gyrus, plausibly through RAGE-dependent ROS amplification, inflammatory signalling, and disruption of trophic support [71,72]. In line with this, AGEs have been reported to impair neural stem cell function and differentiation programs in experimental settings, supporting the concept that chronic AGEs burden can compromise reparative plasticity [73,74]. Importantly, reports of pro-regenerative RAGE signalling (e.g., enhanced progenitor differentiation) typically arise in acute injury contexts and may reflect ligand-, timing-, and microenvironment-specific responses rather than the chronic AGE-dominant state characteristic of neurodegenerative and neuropsychiatric disorders [75,76]. Thus, RAGE effects are context-dependent: transient activation may support repair after acute injury, whereas sustained AGE-driven signalling tends to be maladaptive. In addition, it has been reported to modulate post-injury inflammatory responses, which is important after acute injury, and regulates the immune response by promoting the elimination of dead cells [68]. RAGE also promotes autophagy, which enables cells to withstand stress and maintain homeostasis, particularly during tissue regeneration [67,68].

**Fig. 2:**
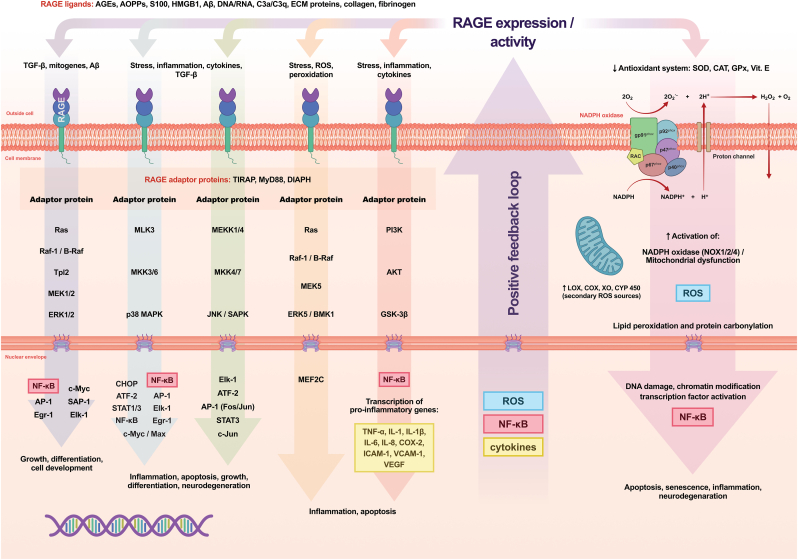
**Overview of receptor for advanced glycation end products (RAGE) signalling pathways and downstream cellular effects.** The receptor for advanced glycation end products (RAGE) responds to a broad and structurally diverse set of ligands. These include advanced glycation end products (AGEs), advanced oxidation protein products (AOPPs), members of the S100 protein family, high mobility group box protein 1 (HMGB1), amyloid-β peptide (Aβ), as well as nucleic acids such as deoxyribonucleic acid (DNA) and ribonucleic acid (RNA). In addition, RAGE can interact with complement components (C3a and C3q) and extracellular matrix (ECM) proteins, including collagen and fibrinogen. Upon ligand engagement, RAGE undergoes conformational changes that facilitate the recruitment of intracellular adaptor proteins. Key adaptors involved in this process include Toll/interleukin-1 receptor domain-containing adaptor protein (TIRAP), myeloid differentiation primary response 88 (MyD88), and diaphanous-related formin-1 (DIAPH1). The formation of these adaptor complexes represents an early step in RAGE-dependent signal transduction. Subsequently, several intracellular signalling pathways are activated. These include the Ras–Raf-1/B-Raf–mitogen-activated protein kinase kinase 1/2 (MEK1/2)–extracellular signal-regulated kinase 1/2 (ERK1/2) cascade, the mixed-lineage kinase 3 (MLK3)–mitogen-activated protein kinase kinase 3/6 (MKK3/6)–p38 mitogen-activated protein kinase (p38 MAPK) pathway, and the mitogen-activated protein kinase kinase kinase 1/4 (MEKK1/4)–mitogen-activated protein kinase kinase 4/7 (MKK4/7)–c-Jun N-terminal kinase/stress-activated protein kinase (JNK/SAPK) axis. RAGE activation also engages the phosphoinositide-3-kinase (PI3K)–protein kinase B (AKT)–glycogen synthase kinase-3β (GSK-3β) pathway. Signals generated by these cascades converge at the level of transcriptional regulation. Prominent downstream effectors include activator protein-1 (AP-1), nuclear factor kappa-light-chain-enhancer of activated B cells (NF-κB), signal transducer and activator of transcription (STAT), and c-Jun. Through these transcription factors, RAGE signalling influences cell proliferation, inflammatory gene expression, and stress-adaptive responses, including the induction of autophagy. When RAGE stimulation is sustained or excessive, additional pathogenic mechanisms become engaged. Increased activity of nicotinamide adenine dinucleotide phosphate oxidase (NADPH oxidase), together with mitochondrial dysfunction, results in elevated production of reactive oxygen species (ROS). At the same time, endogenous antioxidant systems, such as superoxide dismutase (SOD), catalase (CAT), glutathione peroxidase (GPx), and vitamin E (Vit. E), become progressively compromised. These redox imbalances drive downstream oxidative and inflammatory events. Lipid peroxidation and protein carbonylation are enhanced, while the expression of pro-inflammatory cytokines—including tumour necrosis factor-α (TNF-α), interleukin-1β (IL-1β), interleukin-6 (IL-6), and interleukin-8 (IL-8)—is upregulated. In parallel, adhesion molecules such as intercellular adhesion molecule-1 (ICAM-1) and vascular cell adhesion molecule-1 (VCAM-1), as well as vascular endothelial growth factor (VEGF), are induced. Together, these processes establish a self-perpetuating feedback loop that maintains chronic inflammation. Over time, this pathological signalling environment contributes to chromatin modifications, cellular senescence, activation of apoptotic pathways, and the progression of neurodegenerative changes.

RAGE is structurally characterised as a transmembrane protein belonging to the immunoglobulin superfamily [18]. RAGE undergoes oligomerization, forming a functional complex [77]. RAGE contains three immunoglobulin-like domains within its extracellular region. Of these, the V domain is primarily responsible for ligand recognition and binding, including AGEs, and is termed “V” due to its homology with the variable region of immunoglobulins [77,78]. The C1 and C2 are short extracellular domains that allow RAGE to anchor to the cell membrane. The C1 domain, located immediately downstream of the V domain, supports its stability and proper ligand binding, and together they form the VC1 domain (they fold as a unit, which ensures high thermal stability and solubility of the protein) [79]. The alkaline nature of the VC1 contact surface facilitates the interaction of RAGE with ligands, which are typically acidic. The C2 domain is located closest to the cell membrane (downstream of VC1) and stabilises the receptor structure. Both have mainly structural functions [80]. RAGE also contains a single transmembrane domain and a short cytoplasmic tail [18,80]. It is hydrophobic, allowing RAGE to anchor in the lipid layer of the cell [52]. In contrast, the cytoplasmic portion of RAGE does not exhibit kinase activity. Thus, it cannot directly phosphorylate proteins, as is the case for many kinase-coupled transmembrane receptors. Consequently, RAGE must bind to adaptor proteins, e.g., adaptor protein related to the TIR domain (TIRAM, also known as TIRAP) [81], myeloid differentiation primary response 88 (MyD88) [82], formyl peptide receptors (FPRs) [83], or leukotriene B4 receptor 1 (BLT1) [84], which mediate signals into the cell (altering the spatial conformation of RAGE) [18,85] (Fig. 2).

RAGE exhibits broad ligand recognition capacity. The most important ligands of RAGE include AGEs [86], advanced oxidation protein products (AOPPs) [37], calcium-binding protein S100 calgranulins (e.g., S100A12 and S100B) involved in inflammation [66,67,87], high-mobility group box 1 (HMGB1) protein—a chromatin-associated protein which, when released into the extracellular space, acts as an inflammatory mediator [[88], [89], [90]], Aβ—the main component of amyloid plaques [12,91], fibrinogen—a blood protein involved in coagulation [90], DNA and ribonucleic acid (RNA)—nucleic acids released as a result of cell necrosis or damage [13,92,93], some extracellular matrix (ECM) proteins, such as tenascin-C and collagens [94], LPS—components of the cell walls of Gram-negative bacteria [95]. Although the anaphylotoxin fragment C3a (complement component 3a) and C1q (complement component 1q) are not typical RAGE ligands, it has been suggested that they may interact with RAGE in the induction of inflammation [96,97]. The heat shock protein 70 (HSP70) has also been proposed as a potential ligand for RAGE, particularly in the context of inflammatory signalling and cellular stress responses [98,99]. Beyond endogenous ligands, RAGE has also been implicated in responses to selected pathogen-associated molecular patterns (PAMPs) and related inflammatory signals [100,101]. In this context, protein glycation emerges as an important pathogenic mechanism implicated in neurodegeneration [1,17] (Fig. 2).

Ligand binding to RAGE has been shown to activate the mitogen-activated protein kinase (MAPK) pathway, including in neuronal cells [18,80]. MAPKs belong to a family of serine/threonine kinases, and their signalling cascade is organized as a three-tier module in which MAP kinase kinase kinase (MAPKKK) activates MAP kinase kinase (MAPKK), ultimately resulting in MAP kinase (MAPK) activation [102]. Ultimately, this leads to the phosphorylation of the relevant target proteins, resulting in an intracellular response to the signal from the extracellular environment [103]. These kinases are involved in many cellular signalling pathways, determining a specific response depending on the source of activation [89,102,103]. Activation of the extracellular signal-regulated kinase 1/2 (ERK1/2, MAPK) pathway facilitates the initiation of cell differentiation and proliferation, particularly in response to growth factors such as epidermal growth factor (EGF), and also as a result of RAGE activation, with which the ERK1/2 pathway is functionally linked [18]. Activation of the ERK1/2 MAPK pathway has been shown in smooth muscle cells [89], myofibroblasts, osteoblasts, myoblasts [104], and monocytes [105]. This pathway is also found in neural tissue in the brain [106]. Aβ–RAGE interactions in AD have been linked to dysregulation of ERK1/2 MAPK signalling and downstream oxidative–inflammatory stress responses in neurons, which contribute to their degeneration and apoptotic death [107]. Studies have shown that RAGE stimulation is also responsible for the activation of other MAPK family kinases, such as mitogen-activated protein kinases (p38 MAPK) or c-Jun N-terminal kinases/stress-activated protein kinases (JNK/SAPK) [108]. The ERK1/2 MAPK pathway most often promotes cell survival and proliferation [107,109]. In contrast, the p38 MAPK and JNK/SAPK pathways can initiate apoptosis in response to cellular stress, which is crucial in eliminating damaged or malfunctioning cells [102,109]. Upon ligand binding, RAGE can stimulate the *diaphanous-related formin 1 (DIAPH1)* gene, which rearranges the actin cytoskeleton, a process crucial for cell shape, movement, and division [110]. After activation by RAGE, *DIAPH1* promotes actin filament polymerization, which supports cell migration, a process crucial in wound healing as well as tumour invasion. In addition, DIAPH1 contributes to mitosis by forming the mitotic spindle [110,111]. Another signalling pathway, phosphatidylinositol 3-kinase/protein kinase B (PI3K/AKT) activated by RAGE, which promotes cell survival by inactivating pro-apoptotic proteins, supports cell proliferation and growth through the mechanistic target of rapamycin (mTOR) pathway, and also affects glucose metabolism by increasing its uptake through glucose transporter type 4 (GLUT4), which is important in metabolic disorders [112]. It also interacts with TIRAM, which initiates an immune response [113,114]. NF-κB expression is upregulated by these pathways and plays a central role in coordinating cytokine-mediated inflammation, activating adaptive immune responses (B and T cells), promoting cell survival and proliferation via anti-apoptotic mechanisms, and inducing ROS overproduction [115]. Physiologically, NF-κB is inactive in the cytoplasm, bound to the inhibitor of kappa B alpha (IκBα) [114,115]. AGEs, together with other inflammatory stimuli, induce NF-κB activation, which subsequently translocates to the nucleus and drives transcription of genes encoding pro-inflammatory cytokines (e.g., IL-1, IL-6, TNF-α) as well as adhesion molecules such as VCAM-1 and ICAM-1 [52,113,114]. It has been demonstrated that sites where AGEs accumulate excessively also exhibit increased RAGE expression, induction of oxidative stress, and escalation of the inflammatory response [113,116,117]. RAGE activation may also indirectly activate NADPH oxidase 1 (NOX1), NADPH oxidase 2 (NOX2), and NADPH oxidase 4 (NOX4) through various signalling cascades, such as NF-κB, Ras-related protein p21 (p21RAS), MAPK, and PI3K/AKT, including through the activation of protein kinase C (PKC) or the increased expression of proteins regulating NADPH oxidase activity, such as cytochrome *b*-245 light chain (p22phox), neutrophil cytosolic factor 1 (p47phox), and neutrophil cytosolic factor 2 (p67phox), and Ras-related C3 botulinum toxin substrate 1 (Rac1) [118,119]. Large amounts of superoxide radical anion (O_2_•^-^) are generated following NOX activation; this primary ROS is further transformed into other reactive species that induce cellular damage and dysfunction [120]. Additional ROS production from NOX can disrupt mitochondrial function, leading to further oxidative damage, particularly in endothelial cells, heart tissue, and neurons [95,121]. Importantly, ROS production under these conditions is mediated by a positive feedback mechanism—AGEs activate RAGE, which triggers ROS overproduction, and the resulting ROS enhance RAGE signalling [122]. This has been observed, for example, in diabetic retinopathy and neurodegenerative diseases [34,35,123,124]. These observations suggest potential therapeutic directions. It would be reasonable to use antioxidants to inhibit NOX activity in order to mitigate the harmful effects of RAGE activation (Fig. 2).

Physiological expression of RAGE in cells (including neurons) is low. Cellular stress leads to an increase in the concentration of ligands for RAGE. Upregulation of RAGE biosynthesis-related genes occurs in response to oxidative and nitrosative stress and inflammation, particularly in autoimmune and inflammatory diseases such as diabetes, rheumatoid arthritis [125], AD or schizophrenia [76,113,126]. In patients with these diseases, an AGE-dependent increase in RAGE expression has been observed in the endothelium and smooth muscle cells. This results in continuous cellular activation and irreversible tissue damage [112,127,128]. Consistent with the pathways outlined above, carbonyl and oxidative/nitrosative stress form a self-amplifying loop (Fig. 2).

Beyond the canonical membrane-associated receptor, RAGE also exists in soluble circulating forms that lack transmembrane and cytoplasmic regions. These include an endogenously secreted variant (esRAGE) and a proteolytically generated form (cRAGE), both of which retain extracellular ligand-binding capacity [78]. The esRAGE and cRAGE isoforms bind to RAGE ligands, which prevents them from interacting with the membrane-bound form of RAGE, thus limiting receptor activation at the cell surface and reducing pro-inflammatory and pro-oxidant signalling. These soluble isoforms can act as decoy receptors by sequestering RAGE ligands [78]. The ligand binding sites of esRAGE and cRAGE are specific decoy receptors that may be used to inhibit atherosclerosis and neurodegeneration [129]. Although the soluble receptor for AGEs (sRAGE) exhibits promising antagonistic activity as a decoy receptor that neutralises RAGE ligands, the available evidence supporting its therapeutic potential derives from preclinical studies, including cellular and animal models. Consequently, translating sRAGE-based strategies into clinical practice will require overcoming key challenges related to pharmacokinetics, targeted delivery, safety, and a more comprehensive understanding of sRAGE regulation and its pathophysiological role in human diseases [130]. Therefore, interventions that inhibit membrane RAGE activation/expression and/or increase circulating sRAGE (esRAGE/cRAGE) availability have been proposed as potential strategies to enhance decoy-mediated ligand sequestration [131].

## 4 Interaction between AGEs and redox imbalance

Oxidative/nitrosative stress and carbonyl stress form a self-amplifying loop in the brain and the neurovascular unit, in which ROS/RNS promote AGEs formation, while activation of the AGE–RAGE axis secondarily enhances ROS/RNS generation and NF-κB-dependent inflammatory signalling [132,133]. Beyond promoting AGEs formation, ROS may further sustain inflammation through reciprocal amplification involving mitochondrial ROS, NADPH oxidase crosstalk, endothelial nitric oxide synthase uncoupling, and inflammatory cell recruitment, thereby reinforcing NF-κB-dependent cytokine signalling and tissue injury [[134], [135], [136]]. Under conditions of metabolic stress, key ROS (O_2_•^-^, hydrogen peroxide (H_2_O_2_), hydroxyl radical (•OH)) and RNS (nitric oxide (NO), peroxynitrite (ONOO^−^)) promote glycoxidation and glucose auto-oxidation, thereby increasing the pool of reactive dicarbonyls (e.g., MGO) and the accumulation of AGE/ALE adducts [132,137]. Binding of AGEs to RAGE activates NADPH oxidases (NOX1/2/4) and mitochondrial ROS production (electron transport chain (ETC) complexes I and III), increasing O_2_•^-^ and H_2_O_2_ generation and inducing inducible nitric oxide synthase (iNOS) expression. The reaction of O_2_•^-^ with NO leads to the formation of ONOO^−^, which oxidizes and nitrates proteins, promotes tetrahydrobiopterin (BH_4_) oxidation and endothelial nitric oxide synthase (eNOS) uncoupling, and further amplifies oxidative–nitrosative stress and AGE–RAGE signalling [128,138]. ROS/RNS converge on a limited set of molecular targets: proteins (carbonylation, nitration, AGE/ALE cross-linking, and tau/Aβ aggregation) [[139], [140], [141]], lipids (peroxidation of polyunsaturated fatty acids (PUFAs), formation of MDA and 4-HNE) [[142], [143], [144]], mitochondria (mitochondrial DNA (mtDNA) and ETC damage with increased mitochondrial ROS (mtROS) production) [145,146], and the BBB (destabilisation of tight junctions) [145,146]. The dominant sources of ROS/RNS are context-dependent: mtROS and microglial NOX2 in neurodegenerative disorders [147,148], mtROS and NO/ONOO^−^ in depression and chronic stress [[148], [149], [150], [151]], and, in metabolic disorders, a coupling of carbonyl stress with dysregulation of the NOX–NOS axis and activation of AGE–RAGE signalling [149,152] (Table 1). However, evidence in this area remains limited, and further research in human systems is needed.

**Table 1:** Dominant reactive oxygen species (ROS) and reactive nitrogen species (RNS), their cellular sources, and principal molecular targets linking carbonyl, oxidative, and nitrosative stress in neuroinflammatory and neuropsychiatric disorders.

Context	Dominant ROS/RNS	Primary cellular sources of ROS/RNS (brain/neurovascular unit)	Key molecular targets	Functional consequences relevant to glycoxidation and AGE–RAGE coupling	References
**Alzheimer's disease (AD), Parkinson's disease (PD), and other neurodegenerative disorders**	Superoxide anion (O_2_•^-^), hydrogen peroxide (H_2_O_2_), hydroxyl radical (•OH), nitric oxide (NO), peroxynitrite (ONOO^−^; formed from O_2_•^-^+ NO)	Neuronal mitochondrial electron transport chain (ETC) complexes I/III; microglial NADPH oxidase 2 (NOX2); astrocytic redox enzymes; inducible nitric oxide synthase (iNOS) in activated glia	Synaptic proteins; mitochondrial DNA (mtDNA) and respiratory complexes; aggregation-prone substrates (amyloid beta (Aβ), tau, alpha-synuclein (αSyn)); membrane lipids rich in polyunsaturated fatty acids (PUFAs)	Mitochondrial reactive oxygen species (mtROS) and NOX2-derived ROS support carbonyl stress and advanced glycation end products (AGEs) formation; ONOO^−^ drives nitration/oxidation, proteostasis failure, and receptor for advanced glycation end products (RAGE)-linked inflammation	[132,[153], [154], [155], [156]]
**Metabolic syndrome/diabetes-associated brain injury; liver–brain axis**	O_2_•^-^, H_2_O_2_, NO, ONOO^−^	Endothelial NOX–NOS imbalance; mtROS; systemic metabolic sources influencing brain endothelium and microglia	Endothelium/blood–brain barrier (BBB) proteins; inflammasome-related nodes; mitochondrial proteins	Hyperglycemia and carbonyl stress accelerate AGEs formation; AGE–RAGE activation increases NOX and mtROS and promotes inflammatory mediators, sustaining a feed-forward loop	[149,157,158]
**Shared neurovascular mechanism (cross-cutting)**	ONOO^−^; secondary lipid peroxidation aldehydes such as malondialdehyde (MDA) and 4-hydroxynonenal (4-HNE)	NOX and NOS co-activation; endothelial nitric oxide synthase (eNOS) uncoupling via tetrahydrobiopterin (BH_4_) oxidation; lipid peroxidation cascades	Proteins (nitration/carbonylation); membrane lipids (PUFAs); endothelial tight junction proteins	Peroxynitrite chemistry links oxidative and nitrosative injury; eNOS uncoupling amplifies O_2_•^-^, aggravating endothelial dysfunction and BBB permeability	[[159], [160], [161], [162], [163]]

**Table Abbreviations: AGEs**, advanced glycation end products; **AD**, Alzheimer's disease; **PD**, Parkinson's disease; **Aβ**, amyloid beta; **αSyn**, alpha-synuclein; **BBB**, blood–brain barrier; **BDNF**, brain-derived neurotrophic factor; **BH_4_,** tetrahydrobiopterin; **ETC**, electron transport chain; **iNOS**, inducible nitric oxide synthase; **eNOS**, endothelial nitric oxide synthase; **MDA**, malondialdehyde; 4-**HNE**, 4-hydroxynonenal; **mtDNA**, mitochondrial DNA; **mtROS**, mitochondrial reactive oxygen species; **NO**, nitric oxide; **NOS**, nitric oxide synthase; **NOX**, NADPH oxidase; **ONOO^−^**, peroxynitrite; **O_2_•^-^**, superoxide anion; **H_2_O_2_,** hydrogen peroxide; **•OH**, hydroxyl radical; **PUFAs**, polyunsaturated fatty acids; **RAGE**, receptor for AGEs.

Mitochondrial dysfunction and mitochondrial damage-associated molecular patterns (mtDAMPs) are increasingly recognised as key, upstream, and self-propagating mechanisms driving neuroinflammation and neurodegeneration, as well as activation of the AGE–RAGE axis. Mitochondria, as the primary source of ROS and a central regulator of cellular homeostasis, are simultaneously highly vulnerable to oxidative stress–induced damage. Impairment of mitochondrial quality control, including defective mitophagy, leads to loss of mitochondrial integrity and the release of mtDAMPs, such as mtDNA, cardiolipin, adenosine 5′-triphosphate (ATP), and cytochrome *c*, as well as overproduction of mtROS [164,165]. Released mtDAMPs are sensed by pattern recognition receptors (PRRs), including Toll-like receptors (TLRs), resulting in activation of NF-κB signalling, secretion of IL-1β and IL-18, and broad production of pro-inflammatory cytokines and chemokines by microglia and astrocytes [166,167]. Numerous neurodegenerative models demonstrate that mitochondrial dysfunction and mtDAMPs frequently precede overt neuroinflammation and contribute to disease progression by sustaining chronic inflammatory activation [165,166].

Activation of the AGE–RAGE axis further exacerbates mitochondrial pathology by inducing oxidative stress, disrupting mitochondrial function, and upregulating pro-inflammatory mediators in the CNS. In AD models, RAGE-TXNIP (thioredoxin-interacting protein) signalling promotes trafficking of Aβ to microglial mitochondria, leading to increased mtROS production, dynamin-related protein 1 (Drp1)–dependent mitochondrial fragmentation, activation of the NOD-like receptor family, pyrin domain-containing 3 (NLRP3) inflammasome, and release of IL-1β [168]. Consequently, AGE–RAGE signalling and mtDAMPs form positive feedback loops in which RAGE-dependent ROS generation aggravates mitochondrial damage, while mtDAMPs sustain chronic neuroinflammation and neurodegeneration [164,165,168].

**Fig. 3:**
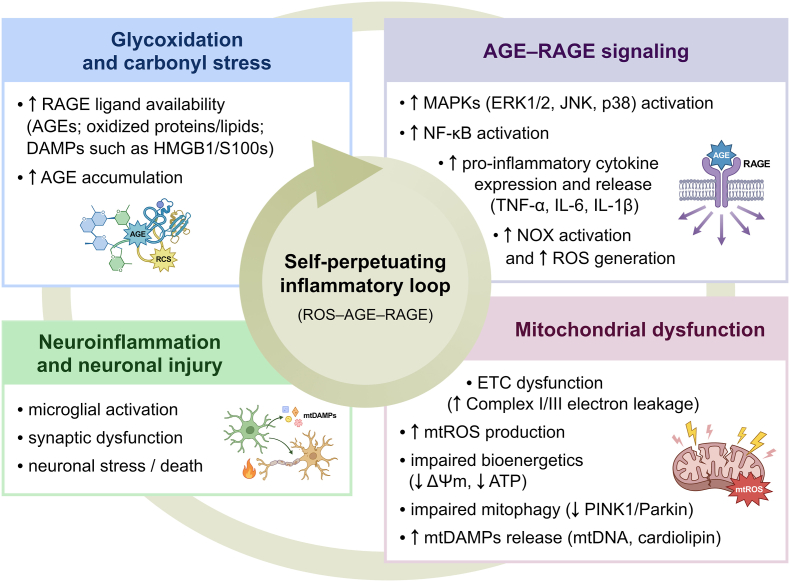
**Integrated interplay between glycoxidation, advanced glycation end products (AGEs)–receptor for advanced glycation end products (RAGE) signalling, mitochondrial dysfunction, and neuroinflammation in the brain.** Glycoxidation and carbonyl stress increase the accumulation of AGEs and other RAGE ligands. These include oxidised proteins and lipids, as well as damage-associated molecular patterns (DAMPs), such as high-mobility group box 1 (HMGB1) and S100 proteins. Binding of these ligands to RAGE activates multiple intracellular signalling pathways, including mitogen-activated protein kinases (MAPKs) like extracellular signal-regulated kinase 1/2 (ERK1/2), c-Jun N-terminal kinase (JNK), and p38 mitogen-activated protein kinase (p38). It also triggers nuclear factor kappa B (NF-κB) signalling. These activations lead to increased expression of pro-inflammatory cytokines such as tumour necrosis factor alpha (TNF-α), interleukin-6 (IL-6), and interleukin-1 beta (IL-1β). At the same time, they enhance activation of nicotinamide adenine dinucleotide phosphate oxidase (NADPH oxidase; NOX) and increase the generation of reactive oxygen species (ROS). Together, these processes promote mitochondrial dysfunction, marked by impairment of the electron transport chain (ETC) and increased production of mitochondrial reactive oxygen species (mtROS). This results in bioenergetic failure, reduced mitochondrial membrane potential (ΔΨm), decreased adenosine triphosphate (ATP) production, and impaired mitophagy linked to reduced PTEN-induced kinase 1 (PINK1) and Parkin RBR E3 ubiquitin protein ligase (Parkin) activity. Mitochondrial damage can also cause the release of mitochondrial-derived damage-associated molecular patterns (mtDAMPs), including mitochondrial DNA (mtDNA) and cardiolipin, which further amplify inflammatory signalling. These mechanisms promote microglial activation, synaptic dysfunction, and neuronal injury. The interactions between carbonyl stress, oxidative stress, AGE–RAGE signalling, mitochondrial dysfunction, and inflammatory pathways create a self-perpetuating pathogenic loop. This loop sustains oxidative stress and chronic neuroinflammation in neurodegenerative and neuropsychiatric disorders.

The relationship between AGE–RAGE signalling and mitochondrial dysfunction appears to be bidirectional and self-amplifying. RAGE activation enhances ROS production and inflammatory signalling, leading to impaired mitochondrial bioenergetics, whereas damaged mitochondria increase oxidative stress and AGEs formation, thereby further potentiating RAGE activation. As a result, a reinforcing pathogenic loop emerges (AGEs/RAGE → ROS/inflammation → mitochondrial dysfunction → increased ROS/AGEs → further RAGE activation), which has been observed in AD and other neurodegenerative disorders [133]. This bidirectional and self-perpetuating interplay between mitochondrial dysfunction, AGEs accumulation, AGE–RAGE signalling, and neuroinflammation is schematically summarised in Fig. 3.

## 5 AGEs and neuropsychiatric disorders

Before discussing individual disorders separately, it is useful to outline several cross-cutting patterns that recur in the available experimental and clinical literature. AGEs are most commonly described as early modulators of the cerebral redox-inflammatory milieu during the initiation phase of neurodegeneration, potentially preceding the emergence of clinically overt neurodegenerative changes. Their gradual accumulation in neurons, glial cells, astrocytes, and the cerebral endothelium—facilitated by ageing and disturbances in glucose metabolism—is consistently observed at preclinical stages, although their direct causal involvement in disease initiation remains a matter of debate [47,169,170]. In biochemical and experimental models, glycation-induced modification of aggregation-prone proteins, including Aβ, tau, αSyn, and prion proteins, is associated with an increased propensity for β-sheet conformations, oligomerization, and the formation of aggregation-seeding structures [169,171]. Concurrently, activation of the AGE–RAGE axis has been linked to NOX engagement, enhanced ROS generation, and NF-κB signalling, thereby favouring the maintenance of a pro-oxidative and pro-inflammatory environment without conclusively establishing the directionality of causative relationships [164,169]. In addition, AGE-associated alterations in endothelial function and BBB integrity correlate with impaired Aβ clearance and increased vulnerability to secondary injurious stimuli, suggesting that AGEs act as one of several modulatory factors shaping early pathological processes [169,171]. However, most human studies are cross-sectional and susceptible to confounding (e.g., metabolic comorbidity, diet) and reverse causation; therefore, temporality and causal direction remain incompletely resolved [172].

During the chronic phase corresponding to neurodegenerative progression, AGEs are more frequently viewed as elements that sustain and amplify ongoing pathological cascades rather than as primary etiological drivers. Continued glycation of Aβ and tau in experimental and post-mortem studies has been associated with increased resistance to proteolytic degradation, enhanced neurotoxicity, and stabilisation of protein aggregates, showing correlations with disease severity [23,169,171]. At the same time, AGE–RAGE signalling operates within a positive feedback framework in which oxidative stress and inflammation promote further AGEs formation, while AGEs are linked to augmented ROS production, NF-κB activation, increased expression of pro-inflammatory cytokines (IL-6, TNF-α), and polarisation of microglia toward an M1-like phenotype, predominantly in preclinical models [16,39,169,173]. Sustained activation of this axis has also been associated with mitochondrial dysfunction, synaptic impairment, neuronal apoptosis, and BBB disruption, although the temporal relationships and hierarchical organization of these events remain only partially resolved [133,169,171,174]. Clinically, elevated AGEs levels measured in blood or skin are associated with accelerated cognitive decline and an increased risk of dementia, including individuals without diabetes, supporting their role as biomarkers and potential contributors to disease progression rather than definitive causal agents [4,132,133]. Consequently, even in the progression phase, the causal direction and the event hierarchy remain difficult to disentangle in humans. Importantly, mechanistic inference relies largely on experimental models, while most human data are cross-sectional and prone to confounding and reverse causation.

### 5.1 AGEs in the pathogenesis of dementia

AD is the predominant cause of dementia in old age [1,175], a condition marked by deterioration of higher cognitive functions, especially short-term and long-term semantic (verbal) memory [2]. Nearly two-thirds of AD patients show PD-associated symptoms and pathological hallmarks, including substantia nigra neurodegeneration and αSyn–dominated Lewy body deposition [176]. Brains of patients with AD exhibit marked atrophy in the cortex and subcortical regions (brain volume decreases by up to 40%) [177]. Certain areas of the CNS—including the temporal, frontal, hippocampal, and amygdalar regions—are additionally affected by localised neurodegeneration, as evidenced by amyloid plaques and neurofibrillary tangles. These plaques are extracellular deposits of Aβ (a peptide consisting of 28 extramembrane amino acids and 11 to 14 hydrophobic residues), surrounded by residual degenerated axons and glial cells [178]. Aβ is formed from amyloid precursor protein (APP), which is encoded by a gene situated on chromosome 21. Glycation of APP has been shown to promote irreversible cross-linking of Aβ, whose molecules form covalent bonds. This mechanism may contribute to neurotoxic pathways implicated in AD progression [179]. Genetic factors play a crucial role in the aetiopathogenesis of AD, particularly through the *APOE ε4* allele, the strongest genetic risk factor for late-onset AD [2,176]. The apoE protein, especially the apoE4 isoform, has been shown to promote Aβ aggregation. These effects are attributed to isoform-specific amino acid substitutions (cysteine (Cys)/Arg at positions 112 and 158), which alter protein conformation and stability. In this context, glycation-derived modifications, including AGEs, may further enhance covalent cross-linking of Aβ aggregates, contributing to amyloid plaque formation [180].

AGEs may promote amyloidogenic APP processing by upregulating key enzymes involved in this pathway, including β-site APP cleaving enzyme 1 (BACE1) and presenilin 1 (PS1), thereby increasing Aβ formation [181,182]. In parallel, carbonyl stress may contribute to tau hyperphosphorylation and aggregation, partly via RAGE-related signalling, further amplifying neurodegenerative changes [[183], [184], [185]]. Taken together, glycoxidation may act less as an isolated initiating event and more as an amplifier of Aβ accumulation, tau pathology, and progressive neurodegeneration [4,183,184].

Under physiological conditions, tau glycation appears low, whereas AGEs modification is enriched in pathological aggregates and increases under carbonyl stress. Although glycation is increasingly recognised as an important post-translational modification of aggregation-prone proteins, direct quantitative interactions between glycation and other post-translational modifications, such as acetylation or ubiquitination, remain poorly characterised and represent an important gap in current knowledge [186].

Other proteins are also involved in the pathogenesis of AD. Paired helical filaments (PHFs), formed from p-tau following microtubule disruption, reflect tau hyperphosphorylation–induced neurofibrillary degeneration, whose density correlates with symptom severity [183,184]. P-tau becomes resistant to proteolytic enzymes. It has been suggested that disulphide bridge formation and tau phosphorylation render tau more susceptible to binding to AGEs [187]. In addition, AGEs are prevalent in neuronal PHFs in AD [188] and are associated with reduced synaptic strength, synapse elimination, and neurodegeneration, notably involving glutamatergic projections to the hippocampus and cerebral cortex [176]. These structural and biochemical alterations extend beyond cytoskeletal pathology and directly affect synaptic function. Importantly, AGEs impair synaptic plasticity and neurotransmission not only through nonspecific oxidative damage but via defined molecular mechanisms, including RAGE–p38 MAPK signalling, dysfunction of synaptic mitochondria, and cytoskeletal destabilisation [174]. In parallel, AGE–RAGE activation interferes with brain-derived neurotrophic factor (BDNF)–tropomyosin receptor kinase B (TrkB) and modulates glutamatergic receptor function, collectively leading to selective impairment of long-term potentiation (LTP), alterations in miniature excitatory postsynaptic currents, and progressive synapse loss [174,189]. These synaptic alterations provide a mechanistic link between carbonyl stress and early cognitive dysfunction preceding overt neurodegeneration [189,190].

The emergence of glycated neuropathogenic proteins in the CNS activates glial cells (astroglia and microglia), triggering inflammation [191]. AGE–RAGE signalling biases microglia toward a pro-inflammatory M1-like state (e.g., via NF-κB/NLRP3-linked inflammatory programs) [192,193]. This can, in turn, induce neurotoxic A1 astrocytes [194] and reduce astrocyte-mediated neuroprotection [195], thereby accelerating age-related neuroinflammation and neurodegeneration [196]. This promotes NF-κB-dependent production of ROS, interleukins, chemokines, and cytotoxic NO [197]. Protein modification and cross-linking are most pronounced in the presence of MGO, which accumulates in tissues in response to ROS [198]. The emergence of degenerated structures reduces proteasome peptidase (PSM) activity, triggering neuronal apoptosis [175]. Imbalances in essential metal ions—including iron, zinc, and copper—have been identified as contributing factors in the development of AD [199]. The BBB regulates the transport of metals; however, in patients with AD, their concentrations in the brain are significantly higher [[200], [201], [202]]. AGEs create preferential binding sites for redox-active metals, particularly iron and copper ions, which act as catalytic centres for oxidative reactions and intermolecular cross-linking, thereby promoting stable protein aggregation. Zinc ions can further modulate aggregation kinetics and aggregate morphology in a context-dependent manner [203,204]. Aβ binds to metals such as iron and copper, reducing them [205]. These reactions produce H_2_O_2_, which is involved in the Fenton reaction, leading to the formation of highly reactive •OH [91,206]. Increased metal ion bioavailability enhances ROS production, leading to an imbalance between secretases and phosphatases, each of which is associated with the formation of new Aβ and tau proteins [207]. Here, NF-κB contributes to the control of oxidative stress and inflammatory signalling under these conditions [91].

An additional AGE-related mechanism contributing to AD pathogenesis involves activation of apoptotic signalling in neurons. Interaction of AGEs with RAGE engages multiple downstream pathways, including PI3K/AKT signalling—a central component of the PI3K/AKT/mTOR axis—thereby altering the balance between anti-apoptotic proteins (e.g., B-cell lymphoma 2 (Bcl-2)) and pro-apoptotic factors such as Bcl-2–associated X protein (Bax) and the tumour suppressor p53 (p53). These molecular changes culminate in caspase-3 (Casp3) activation, which triggers apoptosis and promotes neuronal loss and neurodegenerative progression [208]. AGEs also trigger endoplasmic reticulum (ER) stress by inducing the expression of proteins such as glucose-regulated protein 78 (GRP78) [209]. ER stress has been shown to further promote apoptotic signalling, a process that is exacerbated by intraneuronal accumulation of Aβ [210]. ER stress may also trigger the mitochondrial apoptotic pathway via calcium ion efflux into mitochondria, leading to cytochrome *c* release and downstream caspases activation [211,212]. Sirtuin 1 (SIRT1) modulates this process by influencing redox balance and Aβ production [213]. AGEs increase SIRT1 expression, which can have two effects: cell protection or cell death. Increased SIRT1 expression may also impact autophagic pathways, thereby reducing the removal of pathological proteins, such as tau, which promotes neurodegeneration [213,214].

In addition, brain insulin resistance, referred to as type 3 diabetes, involves insulin signalling disorders in the CNS, leading to increased oxidative stress, carbonyl stress, and AGEs overproduction, further increasing RAGE activation [215,216]. Insulin signalling disorders in the brain promote the accumulation of ceramides (bioactive lipids classified as sphingolipids that act as mediators of cellular stress), which in turn promote the deposition of Aβ and p-tau [216,217]. Increased ceramide accumulation is observed in the hippocampus (particularly in the CA1 and dentate gyrus regions), the frontal and temporal cortex, amygdala, white matter, and hypothalamus of patients with AD [218], which correlates with the severity of oxidative stress, inflammation, and neuronal damage [216,218,219]. High levels of specific ceramides—namely Cer d18:1/24:1, Cer d18:1/18:0, and Cer d18:1/16:0—are commonly observed and are associated with Aβ accumulation and increased tau hyperphosphorylation [216,[218], [219], [220]]. It should be noted that ceramides independently exacerbate oxidative stress and neuronal apoptosis by activating pro-apoptotic pathways. These mechanisms collectively drive neuronal loss and cognitive decline typical of AD [216,218,219].

RAGE expression differs markedly between AD and PD, both in regional and cellular distribution. In AD, RAGE is broadly upregulated in the hippocampus and cerebral cortex, involving pyramidal neurons, microglia, astrocytes, and cerebral endothelial cells, consistent with prominent neurovascular and inflammatory involvement [221]. In contrast, PD shows a more restricted, predominantly neuronal pattern, with RAGE enrichment mainly in dopaminergic neurons of the substantia nigra and, to a lesser extent, striatal neurons, while robust microglial or endothelial upregulation is less consistently observed [222]. These patterns suggest a widespread neurovascular RAGE signature in AD, rather than a primarily nigrostriatal neuronal RAGE profile in PD [222].

The reviewed evidence most consistently supports glycoxidation as a biologically relevant amplifier of AD progression, although its precise role in the earliest stages of disease remains unresolved [223].

### 5.2 AGEs in the pathogenesis of PD

Globally, PD is the second most common neurodegenerative condition, with approximately 6.3 million affected individuals [224]. Its hallmark pathology involves the gradual degeneration of dopaminergic neurons in the substantia nigra of the brain [224,225]. PD is a hypokinetic movement disorder marked by muscle tremors, stiffness, akathisia (restlessness), and bradykinesia (slowness of movement). Other signs include mask-like facial expression, infrequent blinking, bent spine syndrome, and a shuffling gait [226,227]. In approximately 90% of cases, the cause of the disease cannot be identified, although a rare form of parkinsonism is associated with a genetic mutation. The following genes are most commonly involved in familial PD: *SNCA* (PARK1—alternative designation for the mutation), encoding αSyn; *PRKN* (PARK2), encoding parkin; *UCHL1* (PARK5), encoding ubiquitin C-terminal hydrolase L1; *PINK1* (PARK6), encoding PTEN-induced putative kinase 1 (PINK1); *DJ-1* (PARK7), encoding PD protein 7, also known as DJ-1. The *LRRK2* (PARK8) gene encodes leucine-rich repeat kinase 2 (LRRK2), a protein that plays a crucial role in regulating cellular processes, including autophagy and cytoskeletal function. Mutations in LRRK2 (e.g., *G2019S*; other mutations include *R1441C, R1441G, and Y1699C*) are the most common genetic cause of familial PD and account for 1–2% of sporadic cases and up to 10% of familial cases (especially in Ashkenazi Jewish populations and in North Africa) [225,228]. These mutations lead to kinase hyperactivity (excessive phosphorylation and ROS production), causing neuronal degeneration and the development of PD symptoms [225,229]. These mutations are among the most extensively studied in hereditary PD [230,231].

Inhibitors of mitochondrial complex I, including 1-methyl-4-phenyl-1,2,3,6-tetrahydropyridine (MPTP) and its active metabolite 1-methyl-4-phenylpyridinium (MPP^+^), initiate a sequence of biochemical events that culminate in neuropathological features characteristic of the disease in both humans and experimental animal models [229,232]. Elevated levels of iron associated with neuromelanin are observed in the substantia nigra of PD patients, alongside an approximately 50% decrease in GSH compared with healthy individuals [233]. Such conditions facilitate Fenton chemistry, leading to the conversion of H_2_O_2_ into strongly cytotoxic •OH radicals [227,232]. Studies indicate that PD brains display both increased AGEs accumulation and upregulated RAGE expression. Importantly, RAGE signalling has been linked to the induction of oxidative stress in PD [202,230,232,234]. In parallel with progressive neuronal degeneration, microglia become activated and release inflammatory mediators, either through antigen presentation to CD4^+^ T cells via major histocompatibility complex class II (MHC-II) pathways or through the production of pro-inflammatory cytokines such as TNF-α, IL-1β, and IL-6 [235,236]. αSyn is additionally modified post-translationally through processes such as glycation and glycosylation [237]. These modifications primarily involve one of the 15 Lys residues in the αSyn molecule, resulting in the formation of AGE-modified aggregates [237,238]. The oligomerization of αSyn is a crucial pathological modification considered highly toxic [237,239]. Glycated αSyn, in both monomeric and oligomeric forms, has been implicated in the pathogenesis of PD [237,239]. Monomeric αSyn damages DNA through direct interaction, while cross-linked oligomers evade proteasomal degradation, accumulate in neurons, and promote cell death [202,240]. In addition, both types of glycated αSyn promote ROS formation, which enhances the inflammatory cascade. Glycated αSyn also induces neuroinflammation through the activation of microglia and interaction with RAGE, which in turn activates the transcription factor NF-κB [23,202,240]. Increased RAGE expression in PD fuels an inflammatory loop, promoting further αSyn accumulation and neuronal death [23,[239], [240], [241]]. The products of αSyn glycation with d-glucose are also responsible for the abnormal spatial arrangement of this protein, leading to their subsequent deposition in brain tissue. Glycation products are present at the periphery of Lewy bodies throughout all stages of PD [23,238,240,242]. MGO contributes importantly to PD pathogenesis by reacting with dopamine (DA) to generate the neurotoxic metabolite 1-acetyl-6,7-dihydroxy-1,2,3,4-tetrahydroisoquinoline (ADTIQ), thereby accelerating degeneration of dopaminergic neurons in the brain [234]. Both animal and *in vitro* (cellular) models of PD exhibit elevated concentrations of MGO and ADTIQ, implicating glycation in the promotion of neuronal degeneration [243]. MGO additionally disrupts the ubiquitin–proteasome system (UPS), the primary pathway responsible for the clearance of misfolded αSyn. This results in its aggregation and damage to the proteasome [244]. MGO also damages mitochondria, leading to an increased production of ROS [245,246]. Under stress conditions, glycolytic flux increases, which in turn promotes additional MGO production. This mechanism involves the harmful effects of dysfunctional proteins, such as PRKN and PINK1, which increase MGO production by affecting cellular energy metabolism [232,247,248]. The glyoxalase system, comprising GLO1, GLO2, and AR, detoxifies MGO, thereby reducing glycation toxicity. The activity of GLO1, a key antiglycation enzyme, decreases with age, promoting MGO accumulation [249,250]. The *PARK7* gene, which encodes the DJ-1 protein, promotes AGEs metabolism by modulating nuclear factor erythroid 2-related factor 2 (Nrf2) and may act as a deglycosylase; however, its role in MGO detoxification is still not fully understood. Dysfunction of DJ-1 leads to mitochondrial damage, and MGO levels increase as a consequence [[251], [252], [253]]. Although not yet comprehensively characterised, these mechanisms are likely to contribute to PD-associated neurodegeneration. Further research in this area may help to identify the pathways leading to neuronal loss, thereby advancing our understanding of PD pathogenesis and potentially informing the development of more mechanistically targeted therapeutic approaches [254].

Defects in carbonyl detoxification, including an age-related decline in glyoxalase activity and dysfunction of DJ-1/PARK7-related protective pathways, may further increase MGO burden in PD [249,250,252,254]. Accordingly, current evidence does not indicate that glycoxidation alone initiates PD but strongly supports its role in reinforcing established pathogenic cascades once mitochondrial quality control, proteostasis, and carbonyl detoxification are impaired [[249], [250], [251], [252], [253], [254]].

These processes are particularly relevant in dopaminergic neurons of the substantia nigra, where mitochondrial injury and pro-apoptotic signalling reinforce selective neuronal vulnerability. In contrast to neurodegenerative disorders characterised by protein aggregation, schizophrenia represents a condition in which glycation and oxidative stress–related mechanisms appear to operate in a more diffuse and system-level manner.

### 5.3 AGEs in the pathogenesis of schizophrenia

Eugen Bleuler introduced the term schizophrenia as a combination of Greek schizein and phren, which literally means “splitting of the mind”. Schizophrenia is classified as a psychotic disorder [255]. It presents with a disintegration of thinking and perception, as well as maladaptive and flat, or blunted affect, and cognitive impairments. The disorder affects approximately 1% of the general population (affecting both men and women) [1,175]. It is characterised by a variable course, with a tendency toward relapse or recurrence. The classic Schneiderian first-rank symptoms used to diagnose this disease include hallucinations (e.g., auditory—voices commenting in the third person; cenesthetic hallucinations), delusions of thought insertion, withdrawal, broadcasting, delusions of possession, and affective disorders (affective flattening, rigidity, blunted affect) [255,256]. In Japan, the term salience syndrome has been introduced instead of schizophrenia [257]. The DA hypothesis posits that heightened dopaminergic signalling in the mesolimbic pathway represents a key mechanism underlying the principal symptoms of schizophrenia [258]. There are also alternative theories, such as reduced activity of the glutamatergic system, especially N-methyl-d-aspartate (NMDA) receptor (which is a subtype of glutamatergic receptors) [259]. Attention is also drawn to the hyperactivity of 5-hydroxytryptamine 2 (5-HT2) receptors and the important role of inflammation in the development of schizophrenia symptoms [260,261]. Consistently, elevated levels of inflammatory cytokines have been detected in the cerebrospinal fluid of individuals with schizophrenia, including interleukin-1 (IL-1), interleukin-2 (IL-2), and interferon-α (IFN-α) [260]. Decreased prostaglandin concentrations observed in psychotic disorders have been associated with dysfunction of phospholipase A2 [262]. Overproduction of ROS in neutrophils also correlates with negative symptoms of schizophrenia [263]. In schizophrenia, the available evidence links AGE-related pathology less to a single aggregation-prone substrate and more to a broader interface between carbonyl stress, antioxidant impairment, inflammation, and metabolic vulnerability.

Evidence suggests that AGEs accumulation is involved in the underlying mechanisms of psychotic disorders, with potential relevance for schizophrenia [264]. High circulating concentrations of AGEs have been shown to reduce brain tissue volume [265] and induce inflammation that drives disease symptomatology [261]. Therapies associated with lower AGE burden have been linked to clinical improvement in adults with psychotic disorders, although the direction and specificity of this relationship remain to be established [261,265]. This effect is mediated by AGE–RAGE signalling, which induces the production of pro-inflammatory cytokines in astrocytes, neurons, and microglia [266]. Brain tissue is particularly susceptible to the harmful effects of glycoxidation, especially under conditions of impaired antioxidant defence. Patients with schizophrenia have been found to exhibit reduced antioxidant enzymes activity, such as superoxide dismutase (SOD), glutathione peroxidase (GPx), GLO1, and GLO2 [267]. GLO1 plays a key role in detoxifying reactive dicarbonyls, particularly MGO, thereby limiting AGEs formation. Within the glyoxalase pathway, GLO1 catalyses the initial step of MGO detoxification by facilitating its reaction with GSH, leading to the formation of the unstable intermediate S-d-lactoylglutathione [268,269]. Next, GLO2 converts the resulting product into d-lactic acid, which is harmless and can be easily excreted from the cell [270]. GLO1 activity is reduced by approximately 40–50% in schizophrenia as a result of a frameshift mutation in its encoding gene [271]. Emerging genetic associations involving functional variants in *AGER* (e.g., rs2070600, Gly82Ser) and *GLO1* have been reported in schizophrenia [272] and autism spectrum disorder [273] patients, but their effects appear modest and context-dependent, and any contribution to carbonyl stress or AGE-related pathophysiology remains associative and requires replication and functional confirmation [272,273]. Similar, albeit preliminary, associations have also been reported in other neurodevelopmental phenotypes, suggesting broader carbonyl-stress susceptibility signals. However, it is still unclear whether elevated AGEs levels in psychotic disorders begin in the pre-disease period and are primary to the development of symptoms, or whether the increased formation of AGEs remains elevated throughout the disease and is induced by it [241]. Substance-induced psychotic disorder develops as a result of the use of psychoactive substances such as amphetamine, cocaine, cannabis, or lysergic acid diethylamide (LSD). People with a genetic predisposition to developing psychosis are particularly susceptible to developing these symptoms [274]. Studies in individuals with chronic methamphetamine use suggest that this exposure is associated with cognitive impairment and with induction or exacerbation of psychotic symptoms, while oxidative stress and AGE–RAGE signalling appear to be relevant contributing mechanisms [274]. The use of methamphetamine causes an increase in myeloperoxidase (MPO) activity while weakening the defence mechanisms of the antioxidant system, such as catalase (CAT) and GPx [274,275]. The imbalance causes harmful AGEs to accumulate, which activate RAGE, leading to cell damage and the development of inflammation. These changes have been associated with deterioration in cognitive function, attention, task performance, and verbal fluency, as well as with greater severity of psychotic symptoms [276]. Emerging evidence suggests that interventions aimed at attenuating carbonyl stress, oxidative burden, and AGE–RAGE signalling may offer therapeutic benefits [132,276]. Nutraceuticals or antioxidant drugs, together with a healthy lifestyle and a low-sugar, low-AGE diet, may diminish carbonyl stress and oxidative damage in the brain, thereby reducing the risk of disease development and alleviating disease symptoms [17,241,260].

### 5.4 AGEs in the pathogenesis of depressive disorders

Mental disorders are among the most prevalent chronic conditions worldwide. According to WHO data [8], depression is the second most common cause of illness, disability, or premature death. Approximately 350 million people worldwide have depressive disorders, although these figures appear to be significantly underestimated [277]. Lifetime risk estimates indicate that depressive episodes affect a substantial proportion of the population, with consistently higher prevalence observed among women than men [278]. Depression is more than just sadness; it also includes feelings of emptiness, low self-esteem, anger, hopelessness, guilt, and self-isolation. As one of the leading causes of suicide, depression is responsible for nearly 4000 deaths per day, resulting in approximately one million deaths from complications of depressive disorders each year [279]. Approximately half of affected individuals are estimated to not seek professional medical care or psychotherapy. Between 20% and 30% of patients treated for depression suffer from persistent depressive disorder, and around 30% do not respond to traditional therapies [277,280]. Depression is treated with antidepressant medication, sometimes supplemented with other types of neuropsychiatric drugs to increase the effectiveness of treatment [281].

Contemporary aetiological models of depression are based on the monoaminergic hypothesis, assuming dysfunction in the neurotransmission of DA, serotonin (5-HT), noradrenaline (NA), and acetylcholine (ACh) [282]. Neuroendocrine dysregulation has also been implicated in these conditions, encompassing alterations in hypothalamic–pituitary–adrenal axis activity as well as disturbances in thyroid hormone homeostasis, most frequently manifesting as hypothyroid states [282]. Organic depressive disorders have been associated with structural alterations in the CNS. Furthermore, it has been suggested that immune system disorders, as proposed by the inflammatory hypothesis of depression (e.g., elevated pro-inflammatory cytokines such as TNF-α, IL-1β, and IL-6, along with increased ROS), or epigenetic changes (DNA methylation), may also contribute to an increased risk of developing a depressive episode [[282], [283], [284]]. Despite intensive research, the aetiology of depression is still unclear. For the disease to manifest, specific non-biological factors, including psychological, as well as environmental, genetic, and epigenetic factors, must occur simultaneously [280,283,284]. Within this multifactorial framework, glycation- and oxidative stress–related processes appear to be most relevant as mechanisms linking chronic oxidative stress with impaired neuroplasticity [7,285,286]. Reduced neuroplasticity is a characteristic feature of depression and is accompanied by volume loss in the hippocampus and prefrontal cortex [287]. These alterations are strongly influenced by oxidative stress and accumulation of AGEs that affect CNS function [288,289]. Overproduction of ROS and deficiency or depletion of antioxidant mechanisms may be associated with the accumulation of AGEs, disrupting CNS homeostasis [197]. Multiple studies indicate that oxidative stress and AGEs accumulation participate in the complex pathogenic framework underlying depression [287,290,291]. One study reported that individuals with melancholic depression had the highest levels of AGEs (2.61 mean arbitrary units) compared to individuals with non-melancholic depression (2.45 mean arbitrary units) and individuals without depression (2.38 mean arbitrary units). A statistically significant correlation has also been demonstrated between higher levels of AGEs and depressive symptoms, especially in melancholic depression [290]. Furthermore, the association between AGEs and depression has been demonstrated in the Maastricht Study [7], showing that tissue AGEs burden—assessed non-invasively by skin autofluorescence (SAF)—is associated with greater severity of depressive symptoms [272,273]. This relationship remained significant after adjustment for potential confounders, such as age, sex, (non-insulin-dependent) diabetes, smoking, abnormal body mass index (BMI) or kidney dysfunction. A unit increase in SAF (by 1 standard deviation) was associated with a 42% increase in the risk of depressive disorder. Higher SAF levels correlated with both cognitive (e.g., lack of interest, low mood, concentration problems, suicidal ideation) and somatic (e.g., sleep disorders, fatigue, poor appetite) symptoms of depression, suggesting that the accumulation of AGEs is associated with depression as a whole, rather than just its somatic aspects. Accordingly, plasma levels of Nε-(carboxyethyl)lysine (CEL), Nε-(carboxymethyl)lysine (CML)—both widely used AGEs markers—and pentosidine (PEN) were not significantly associated with depression following confounder adjustment [7]. Excessive AGEs accumulation may secondarily interfere with BDNF/TrkB-related signalling, thereby contributing to impaired neuroplasticity and to hippocampal and prefrontal dysfunction in depression. However, the direct impact of AGEs on this pathway remains insufficiently defined [7,11,289,292].

Studies have shown that polymorphic gene variants involved antioxidant mechanisms, such as genes encoding SOD2, GPx4, and CAT [293], as well as genes associated with nitrosative stress, including nitric oxide synthase 1 and 2 (NOS1, NOS2) [[293], [294], [295]], and genes encoding enzymes of the tryptophan (Trp) catabolic pathway, such as tryptophan hydroxylase 1 and 2 (TPH1, TPH2), indoleamine 2,3-dioxygenase 1 (IDO1), and kynurenine aminotransferase I and II (KATI, KATII) [291,296], may increase the risk of depressive episodes [[293], [294], [295], [296]]. Animals subjected to chronic stress exhibited increased transcriptional activity of the *TPH2* locus, resulting in enhanced 5-HT synthesis. Treatment with venlafaxine, a serotonin–noradrenaline reuptake inhibitor (SNRI), restored *TPH2* expression toward baseline levels [297]. Neuronal dysfunction and neurodegeneration arise in part from oxidative and nitrosative stress–mediated damage to lipids, proteins, and DNA. A higher risk of depression has been reported to be associated with the polymorphism c.47T > C (rs4880) in the gene encoding *SOD2* [293]. Similar relationships have been reported for polymorphisms in the genes encoding *CAT* and *GPX4* (c.-89A > T (rs7943316) and c.660T > C (rs713041)) [298]. Patients with depression had elevated levels of biomarkers of oxidative damage, such as 8-oxoguanine (8-oxoG) and MDA [299]. In addition, disruption of the Trp pathway can also lead to the overproduction of neurotoxic compounds, such as quinolinic acid, which increases ROS production and induces neuronal apoptosis [300]. Stress-related depressive models show increased IDO1 expression and activation of the Trp–kynurenine pathway, changes that are mitigated following antidepressant treatment [301].

## 6 Neurological and psychiatric drugs with antiglycation properties

### 6.1 Antiparkinsonian drugs with antiglycation properties

Currently, the primary goal of PD drug therapy is to alleviate motor and non-motor symptoms (e.g., dementia). Unfortunately, none of the antiparkinsonian drugs effectively halt disease progression and, at best, provide only symptomatic slowing [225,227]. As a precursor of DA, levodopa (l-DOPA) remains widely used in therapeutic regimens, frequently in combination with additional pharmacological agents [225,227]. Treatment also involves the use of dopamine agonists (DAs), monoamine oxidase B (MAO-B) inhibitors, and catechol-*O*-methyltransferase inhibitors (COMTIs). Complementary therapeutic agents are often employed to target non-motor symptom domains, with the aim of improving general functional status [227]. Acetylcholinesterase inhibitors (AChEIs) are used to treat dementia in advanced PD [302]. Antipsychotic drugs (APDs), also referred to as neuroleptics, alleviate hallucinations and psychosis [303], while antiepileptic drugs (AEDs) or antidepressants can be used to treat mood disorders and control impulses [304,305].

Currently, apomorphine is the only PD therapy reported to inhibit glycation in experimental settings [306]. In contrast, bromocriptine has only an indirect beneficial effect on glycation markers, through improved glycaemic control and reduced oxidative stress [307,308]. There are no reliable studies confirming the protective effect of amantadine against glycation; in some systems, it even increases the production of AGEs [309].

Most drugs used to treat PD (levodopa, selegiline, rasagiline, safinamide, pramipexole, rotigotine, pergolide) have only been evaluated in clinical trials for their effects on ROS production, lipid peroxidation, antioxidant enzyme activity, or RAGE expression. The lack of detailed analyses of glycation, glycoxidation, and carbonyl stress represents a significant research gap. In a rotenone mouse model, the combination of levodopa (with carbidopa) and canagliflozin has been reported to modulate signalling events associated with the RAGE–NF-κB axis, alongside favourable effects on mitochondrial functional parameters [310]. Selegiline (together with rasagiline) as a MAO-B inhibitor reduces ROS levels, stabilises mitochondrial function, and activates the Nrf2 pathway [311,312]. Pramipexol (DAs), in a rat model with Aβ1-42 infusion, attenuated redox imbalance and mitochondrial dysfunction and was associated with improved cognitive outcomes [313]. In a mouse model of LPS-induced neuroinflammation, rotigotine (DAs) reduced lipid peroxidation and restores CAT activity and GSH levels [314]. Pergolide (DAs) has been reported to interfere with NF-κB translocation and to mitigate oxidative stress–associated apoptosis in a H_2_O_2_-treated human neuroblastoma (SH-SY5Y) cell model [315]. In contrast, safinamide, an MAO-B inhibitor, in a model of human neuroblastoma cells (M17) exposed to Aβ1-42, reduces ROS production, normalises GSH levels, and activates the protective SIRT1 pathway [316]. Collectively, these observations support a predominantly indirect, redox- and inflammation-modulating mode of action rather than unequivocal direct antiglycation activity. An overview of these agents and their antiglycation potential is provided in Table 2 (partial/indirect evidence), while detailed model-specific findings and expanded mechanistic descriptions are presented in Supplementary Table S1.

**Table 2:** Neurological and psychiatric drugs evaluated for antiglycation-related activity: summary of available evidence, key proposed mechanism, and representative references.

Drug	Has a direct antiglycation effect been demonstrated?	A brief description of the mechanism of action	Citation
**Antiparkinsonian drugs**			
**Amantadine,** increases extracellular dopamine (DA)	No (*in vitro*; possible proglycation effect)	No relevant antiglycation activity; possible proglycoxidative effect	[309]
**Apomorphine**, a potent dopamine agonist (DAs)	Yes (*in vitro*)	Fe^2+^ chelation; reactive oxygen species (ROS) reduction	[306]
**Bromocriptine** (DAs)	Yes, but indirect rationale (*in vivo*)	Carbonyl stress reduction; antioxidant effect	[307,308]
**Drugs used to treat dementia, including Alzheimer's disease (AD)**			
**Memantine,** an N-methyl-d-aspartate receptor antagonist (NMDA)	Yes (*in vitro*)	Carbonyl scavenging; inhibition of Janus kinase 2/signal transducer and activator of transcription 1 (JAK2/STAT1) signalling	[317]
**Galantamine,** an acetylcholinesterase inhibitor (AChEIs)	Yes (*in vivo*)	Alpha7 nicotinic acetylcholine receptor (α7 nAChR) activation; inhibition of JAK2/signal transducer and activator of transcription 3 (STAT3), nuclear factor kappa B (NF-κB), and high-mobility group box 1/receptor for advanced glycation end products (HMGB1/RAGE) signalling; brain-derived neurotrophic factor/tropomyosin receptor kinase B (BDNF/TrkB) activation	[318,319]
**Donepezil** (AChEIs)	Yes (*in vivo*)—partially confirmed, but a proglycation effect is also observed	Reduction of advanced glycation end products (AGEs); antioxidant and anti-inflammatory effects; mixed glycation profile (indicating increased glycation in hyperglycaemia)	[[320], [321], [322]]
**Rivastigmine** (AChEIs)	Yes (*in vivo*)	Reduction of AGEs; sirtuin 1 (SIRT1) activation; attenuation of oxidative stress, NF-κB signalling, and mechanistic target of rapamycin (mTOR) dysregulation	[323,324]
**Drugs used in the treatment of psychotic disorders (schizophrenia)**			
**Olanzapine,** a second-generation antipsychotic (SGAs)	Yes (*in vivo* and *in vitro*)	Inhibition of protein carbonyl formation; attenuation of AGE–RAGE-related signalling	[325,326]
**Clozapine** (SGAs)	No (*in vitro* and *in vivo*), proglycation effect	Increased protein carbonyls, lipid peroxidation, and ROS formation	[[327], [328], [329], [330], [331]]
**Paliperidone**, an active metabolite of risperidone	Yes (*in vitro* and *in vivo*)	RAGE/HMGB1 downregulation; antioxidant neuroprotection	[[332], [333], [334], [335]]
**Risperidone** (SGAs), oral and long-acting injectable (LAI)	Yes (*in vivo*)	Reduction of Nε-(carboxymethyl)lysine (CML); inhibition of AGE–RAGE–NF-κB signalling	[336]
**Haloperidol,** a first-generation antipsychotic (FGAs)	No (*in vivo*), proglycation effect	Increased protein carbonyls and lipid peroxidation	[331,337,338]
**Quetiapine** (SGAs)	No (*in vivo*)—partially confirmed	Mixed evidence; possible antioxidant/neuroprotective effects with potential promotion of glycation	[[339], [340], [341], [342], [343]]
**Drugs used in the treatment of depressive disorders**			
**Agomelatine**, a noradrenaline–dopamine disinhibitor	No (*in vitro*)—shows a pro-AGE effect	No carbonyl-scavenging activity; possible pro-AGE effect	[344]
**Trazodone,** shows a mixed mechanism of action and affects serotonergic conduction	No (*in vitro*)—appears to enhance glycation	Enhanced AGEs and advanced oxidation protein product (AOPPs) formation	[345]
**Paroxetine,** a selective serotonin reuptake inhibitor (SSRI)	Yes (*in vitro* and *in vivo*)—partially confirmed	Indirect attenuation of AGE–RAGE-related damage via reduced mitochondrial ROS and oxidative stress	[346]
**Fluoxetine** (SSRI)	Yes (*in vivo*)—partially confirmed	Modulation of S100 calcium-binding protein B (S100B)/RAGE signalling	[347]
**Escitalopram** (SSRI)	Yes (*in vivo*)—partially confirmed	Indirect suppression of RAGE/NF-κB signalling; improved glycaemic control	[348]
**Vortioxetine** (multimodal serotonin modulator)	Yes (*in vivo*)—partially confirmed	Attenuation of S100B/RAGE/NF-κB signalling	[349]

**Table abbreviations**: **AD**, Alzheimer's disease; **AGEs**, advanced glycation end products; **AOPPs**, advanced oxidation protein products; **BDNF**, brain-derived neurotrophic factor; **CML**, Nε-(carboxymethyl)lysine; **DAs**, dopamine agonists; **DA**, dopamine; **FGAs**, first-generation antipsychotics; **HMGB1**, high-mobility group box 1; **AChEIs**, acetylcholinesterase inhibitors; **JAK2**, Janus kinase 2; **LAI**, long-acting injectable; **mTOR**, mechanistic target of rapamycin; **NF-κB**, nuclear factor kappa B; **NMDA**, N-methyl-d-aspartate; **RAGE**, receptor for AGEs; **ROS**, reactive oxygen species; **S100B**, S100B protein; **SGAs**, second-generation antipsychotics; **SIRT1**, sirtuin 1; **SSRI**, selective serotonin reuptake inhibitors; **STAT1/3**, signal transducer and activator of transcription 1/3; **TrkB**, tropomyosin receptor kinase B; **α7 nAChR**, alpha7 nicotinic acetylcholine receptor.

Currently, the primary measures used to reduce carbonyl stress and AGEs burden in PD patients are indirect, such as blood glucose control, a diet low in AGEs, and antioxidant supplementation. However, there is no drug (with proven efficacy) that combines antiparkinsonian properties with a direct antiglycation effect [350].

### 6.2 Anti-dementia drugs with antiglycation properties

In the absence of disease-modifying cures, treatment strategies for dementia prioritise mitigation of symptom severity and preservation of functional capacity over time, including in AD [351]. In clinical practice, AChEIs, including donepezil-, rivastigmine-, and galantamine-based therapies, are used to improve cognitive function (based on the ACh deficiency hypothesis). In more advanced stages of the disease (particularly in moderate and severe cases), memantine, an NMDA receptor antagonist that modifies glutamatergic projections, is also used [352]. When standard treatment does not produce satisfactory outcomes, AEDs are used to alleviate agitation, aggression, irritability, and emotional instability [353].

Memantine is currently the only approved drug reported to show a direct inhibitory effect on the pro-inflammatory cascade initiated by AGEs in *in vitro* models, with additional carbonyl-scavenging potential [317]. The most effective AChEIs in reducing AGEs are rivastigmine [323,324] and galantamine [318,319], which are superior to donepezil [320,321,323] in this regard. Clinical studies have shown that AD patients treated with rivastigmine had lower serum concentrations of AGEs, which may indicate potential antiglycation-related effects, possibly linked to modulation of the cholinergic system and attenuation of RAGE-related signalling [323,324]. Galantamine not only inhibits AChE activity, but has also been reported to reduce the levels of key glycation biomarkers (CML and Nω-(carboxymethyl)arginine (CMA)), particularly in models of chronic cerebral ischaemia. Additionally, it has been associated with lower RAGE expression and reduced NF-κB activation, supporting a possible neuroprotective effect [318,319]. Donepezil, on the other hand, has a limited ability to affect existing glycation changes, acting mainly by reducing metabolic stress. However, it does not reverse existing AGEs [[320], [321], [322]] and, under hyperglycaemic conditions, may even exacerbate glycation processes, as reflected in inconsistent study findings [321]. Despite the available studies, these drugs require further, more targeted research with the measurement of classic markers of AGEs, such as PEN, argpyrimidine (ARG), and vesperlysine (VES), among others (Table 2).

*In vitro* glycation models indicate that effects of anti-dementia drugs are bidirectional and strongly model-dependent, complicating any a priori assumptions regarding combinatorial “synergy”. Under fructose-driven glycation, donepezil markedly increased ARG and total AGEs, whereas lamotrigine and aminoguanidine reduced selected glycation endpoints; conversely, under MGO-driven conditions, several agents attenuated APs formation, whereas donepezil exerted a pro-glycative effect. Collectively, these *in vitro* patterns are strictly hypothesis-generating and underscore the need for *in vivo* and clinical validation before any translational inference can be made [354].

In anti-dementia pharmacology, the available evidence suggests a model-dependent and sometimes bidirectional pattern in which some agents attenuate selected AGE-related pathways, but the distinction between true antiglycation activity and broader anti-inflammatory or cytoprotective effects remains insufficiently resolved [354].

### 6.3 Antipsychotic drugs with antiglycation properties

Schizophrenia is a disease that requires lifelong medication. APDs are the main medications used to treat acute psychosis and control the course of the disease, limiting its recurrence. APDs have a wider clinical application that extends to schizoaffective and bipolar disorders, as well as to certain depressive presentations and dementia, including AD [355]. In PD, antipsychotics with strong D2 receptor blockade are generally avoided due to the risk of worsening motor symptoms; only a limited number of newer APDs are used to manage psychosis in this context [267]. APDs are divided into typical (first-generation antipsychotics, FGAs) and atypical (second-generation antipsychotics, SGAs). FGAs include chlorpromazine, levomepromazine, perazine, haloperidol, chlorprothixene, flupenthixol, zuclopenthixol, sulpiride and tiapride. More recent SGAs include clozapine, olanzapine, quetiapine, risperidone, paliperidone, ziprasidone, lurasidone, aripiprazole, brexpiprazole, cariprazine, and amisulpride [356,357]. Before summarising compound-specific findings, it should be emphasised that apparent 'anti' versus ‘pro-glycation’ signals in antipsychotics likely reflect differences in receptor-driven metabolic liability and downstream oxidative/carbonyl stress rather than direct inhibition of glycation chemistry. Compounds characterised by prominent H1 and muscarinic (M1/M3) antagonism and greater autonomic liability (e.g., clozapine, olanzapine) are consistently associated with metabolic dysregulation, which may secondarily exacerbate oxidative and carbonyl stress, thereby favouring the accumulation of AGEs [358,359]. In contrast, agents with a comparatively neutral metabolic profile (e.g., aripiprazole, ziprasidone, lurasidone, and in certain clinical contexts risperidone) may impose a lower burden of carbonyl stress and AGE–RAGE-related signalling rather than functioning as primary inhibitors of glycation [360,361].

Studies to date have shown that olanzapine, an SGAs, reduces blood levels of esRAGE, which may indicate an indirect weakening of AGE–RAGE signalling [325]. Additionally, it inhibits the formation of protein carbonyls (PCOs) [326]. Risperidone (SGAs) exhibits antiglycation properties in a mouse model, reducing AGEs (CML) in the hippocampus and inhibiting activation of the RAGE–NF-κB pathway [336]. Haloperidol (FGAs) increases PCOs levels in the hippocampus and enhances lipid peroxidation (increased production of thiobarbituric acid reactive substances (TBARS)), indicating proglycation and pro-oxidation properties [331,337,338]. Paliperidone (SGAs), the active metabolite of risperidone, has been implicated in modulating glycation-related signalling, with reported interactions involving RAGE and concurrent attenuation of RAGE- and HMGB1-associated expression patterns [332]. In addition, it has potential neuroprotective properties resulting from the inhibition of oxidative stress markers [[333], [334], [335]]. Clozapine (SGAs) increases PCOs levels and enhances lipid peroxidation [327], and induces ROS synthesis, confirming its proglycative and pro-oxidant effects [[328], [329], [330], [331]]. There are no direct studies on the effect of quetiapine (SGAs) on AGEs, but even at low doses, an increase in glycated haemoglobin (HbA1c) is observed, which may promote glycation [339]. Prolonged exposure to quetiapine has been associated with favourable shifts in oxidative balance and with cognitive outcomes in neurodegenerative contexts, including AD and PD [[340], [341], [342], [343]]. In parallel, mechanistic insights from cellular models have suggested that quetiapine can influence amyloidogenic processes, as evidenced by reduced aggregation propensity of Aβ in pheochromocytoma-derived PC12 cells (PC12) (AD model) [362]. By promoting a pro-oxidative milieu, zuclopenthixol (FGAs) may facilitate Maillard chemistry and subsequent AGEs formation [363]. In the case of lurasidone (SGAs), no direct data on its effect on AGEs are available; however, it has an indirect protective effect. Chronic treatment with lurasidone activates the Nrf2 pathway (increases its expression and simultaneously decreases the level of Kelch-like ECH-associated protein 1 (KEAP1)), which increases the transcription of carbonyl detoxification enzymes (e.g., CAT) and suppresses the expression and protein levels of NOX2, a major ROS generator in microglia. These effects are associated with reduced oxidative stress in the hippocampus of stressed rats, alongside facilitated carbonyl detoxification and decreased AGEs formation [364]. Similarly, in the case of aripiprazole (SGAs), no direct effect on AGEs production has been investigated. Reduced lipid peroxidation, evidenced by lower MDA and TBARS levels, was observed in Wistar rats and was accompanied by an increase in the *in vivo* activity of antioxidant enzymes, including GPx, GSH, and CAT [365]. These patterns were corroborated by a meta-analysis of investigations conducted in human cohorts [366]. Notably, patients diagnosed with schizophrenia exhibited enhanced antioxidant status in peripheral blood samples. This indicates an indirect anti-AGE effect, which is particularly important in patients at high risk of carbonyl stress (patients with diabetes, smokers) [[365], [366], [367]]. *In vivo* high-dose combination therapy (FGAs + SGAs) correlates with increased PEN levels. In monotherapy (FGAs or SGAs), such a correlation did not emerge [368] (Table 2).

No studies have been conducted on the effect of most classic FGAs and more recent SGAs on the glycation process. Experiments are needed to determine the levels of CML, CEL, or AGEs in people treated with antipsychotic drugs. Currently, there is insufficient evidence that any of the APDs used to treat psychosis have a proven antiglycation activity *in vivo*. Only indirect effects resulting from modifications in oxidative stress are generally observed [366,369].

For antipsychotic drugs, apparent anti-versus pro-glycation effects should be interpreted cautiously, as they are likely to reflect broader differences in metabolic liability, oxidative burden, and inflammatory signalling rather than uniform direct effects on glycation reactions [359,370].

### 6.4 Antidepressants with antiglycation properties

Antidepressants are a diverse group of drugs that are used to treat depressive disorders, anxiety, and other mental health conditions (often accompanied by depression). In terms of their mechanism of action, they primarily affect the neurotransmitter system in the brain, regulating the levels of 5-HT, NA, and DA [281]. The major groups of antidepressants include tricyclic antidepressants (TCAs), monoamine oxidase inhibitors (MAOIs), selective serotonin reuptake inhibitors (SSRI), SNRI and more recent drugs, including bupropion, mirtazapine, nefazodone, trazodone, vilazodone, and vortioxetine [281]. The clinical efficacy of these drugs has been confirmed, but they often cause troublesome side effects, such as sleep disorders, dry mouth, weight gain, sexual dysfunction, and dyspeptic symptoms [280,284]. The balance between efficacy and tolerability of these drugs is a matter of individual patient response, the type of drug used, and the dose [281,371]. An unquestionable advantage is that some new antidepressants, such as vortioxetine and bupropion, improve cognitive function in individuals with depression (by increasing BDNF) [372]. Antidepressants are key to treating mood disorders, but their efficacy and tolerability should be monitored regularly throughout treatment [281,371,372].

Some antidepressants may exhibit additional mechanisms of action. Paroxetine (SSRI) appears to exert mainly indirect antiglycation-related effects through attenuation of hyperglycaemia-driven mitochondrial ROS and RCS production, together with downstream dampening of AGE–RAGE signalling, rather than through a confirmed direct antiglycation mechanism [346]. Furthermore, at the tissue level, it has been reported to improve vascular endothelial function [346]. Paroxetine also has an “upstream” role, reducing the hyperglycaemic production of mitochondrial ROS and RCS, thereby constraining the availability of reactive carbonyl intermediates, including MGO [346]. Importantly, the drug also alleviates the direct effects of AGEs, inhibiting the activity of the AGE–RAGE pathway and reversing AGE-induced damage to podocytes. These measures result in reduced proteinuria, suppression of thickening of the basement membrane in the kidneys, and overall improvement in vascular function, as well as protection against diabetic complications [346,373]. Agomelatine, one of the newer antidepressants acting as a noradrenaline–dopamine disinhibitor, has not been shown to exert a measurable effect on AGEs formation. In bovine serum albumin (BSA)-based glycation assays employing glucose-, fructose-, GO-, or MGO-driven conditions, no significant changes were observed in AGE-related fluorescence signals or RCS readouts compared to the corresponding controls. Moreover, in some conditions, an increase in glycation was even observed compared to the control [344]. Trazodone, a drug with a mixed mechanism of action that modulates serotonergic conduction, exhibits proglycation activity confirmed by *in vitro* studies. Compared to aminoguanidine (a standard compound with antiglycation effects), trazodone was found to be significantly less effective in reducing glycation [345]. Fluoxetine (SSRI) affects esRAGE, but studies directly assessing its effect on AGEs production are lacking. In a chronic mild stress model, it restored S100B and RAGE expression in the hippocampus; however, AGEs levels were not determined [347]. The indirect antiglycation effect of escitalopram (SSRI) has been confirmed in studies in rats with type 2 diabetes. Oral therapy with this drug resulted in improved glycaemic control, as well as reduced myocardial expression of RAGE, NF-κB and pro-inflammatory cytokines. Although no direct measurement of AGEs levels was performed, the reduction in RAGE indicates a weakened cellular response to existing AGEs [348]. Vortioxetine (a multimodal drug) also shows an indirect antiglycation effect. This effect appears to involve modulation of the S100B/RAGE axis and downregulating NF-κB expression [349] (Table 2).

In the case of the most popular antidepressants, such as SSRI (sertraline, fluvoxamine), SNRI (duloxetine), and TCAs, including clomipramine and amitriptyline, there are no studies directly evaluating their effect on the level of AGEs. This apparent gap in the literature highlights the need to include testing of Maillard reaction products in future studies of the pleiotropic effects of antidepressants [374].

Overall, the available literature does not support a class-wide direct antiglycation effect of neuropsychiatric drugs; in most cases, reported benefits are more plausibly explained by indirect modulation of oxidative stress, AGE–RAGE signalling, mitochondrial dysfunction, or metabolic burden than by direct interference with glycation chemistry itself. Accordingly, apparently conflicting findings, including the coexistence of antioxidant and pro-glycation effects, should be interpreted as context-dependent rather than as true mechanistic contradictions. Importantly, no single shared antiglycation pharmacophore emerges across these structurally diverse agents; where more direct chemical effects are plausible, they appear to be associated mainly with redox-active aromatic motifs (e.g., the catechol moiety of apomorphine) and, in selected cases, amine-containing scaffolds with potential carbonyl-scavenging capacity, whereas the broader pattern across drug classes is functional convergence on attenuation of oxidative stress, AGE–RAGE/NF-κB signalling, mitochondrial dysfunction, and metabolic burden rather than direct interference with glycation chemistry itself [375,376].

This limitation substantially constrains therapeutic translation, because indirect redox- or inflammation-modulating effects cannot by themselves be interpreted as evidence of true *in vivo* antiglycation efficacy [136].

## 7 Other potential therapeutic perspectives and AGEs biomarkers

### 7.1 Potential prospects using anti-AGEs properties

One therapeutic approach in neuropsychiatric diseases is the inhibition of microglia activity, which can reduce neuroinflammation. These include NF-κB pathway inhibitors [377], such as Bay 11-7082 (an inhibitor of IκB kinase (IKK), thereby preventing NF-κB pathway activation) [378], minocycline (which reduces IL-6 and BACE1 levels) [379], etanercept (a TNF-α inhibitor) [380], and forsythoside B (which reduces the phosphorylation of IKKα/β, two isoforms of IκB) [381]. These drugs can be used to limit neuronal damage caused by chronic inflammation (which is often accompanied by glycation stress). Such mechanisms are highly relevant to the pathobiology of neurodegenerative disorders, including AD and PD, where loss of cellular homeostasis contributes to progressive neuronal decline. Another important treatment approach is to strengthen the BBB, as its dysfunction leads to brain damage (increased permeability to toxic agents). Agents such as angiopoietins (e.g., angiopoietin 1, which stabilises blood vessels and strengthens tight junctions in the BBB, promoting its integrity) are being investigated. Therapies that modulate pericyte function are under development [382]. Potential drugs include protein C (a natural anticoagulant in blood plasma) [383] and cyclophilin A inhibitors (e.g., cyclosporine A) [384,385], which may improve BBB integrity and thus neurological function [386]. Beyond downstream anti-inflammatory or barrier-stabilising approaches, interest is also growing in small-molecule strategies that can directly modulate RAGE–ligand interactions, although evidence remains preliminary. Most of these strategies target downstream consequences of glycation rather than AGEs formation per se. Exploratory *in silico* analyses and limited *in vitro* observations have suggested that certain FDA-approved compounds, including paliperidone, can associate with RAGE and modulate selected downstream readouts related to RAGE/HMGB1 pathways and glycation. These preliminary findings are hypothesis-generating only and, in the absence of *in vivo* evidence, do not support any inference regarding therapeutic efficacy or clinical relevance [332].

Therapeutic strategies focused on protecting mitochondria, which are particularly vulnerable to damage through protein glycation, are also being considered. In recent years, coenzyme Q10, MitoQ (a modified form of coenzyme Q10 (CoQ10)), and other mitochondrial antioxidants such as resveratrol [387], and N-acetylcysteine (NAC) [388] and have been studied. Available evidence suggests that such compounds may influence neuronal survival by shaping oxidative homeostasis and apoptotic signalling, rather than by directly preventing cell death [389,390]. Autophagy, particularly the sequestosome 1–dependent pathway (p62/SQSTM1), constitutes a key lysosomal mechanism for the clearance of AGE-modified and glycoxidatively damaged proteins, thereby preserving cellular proteostasis [391,392]. Chronic AGEs overload can impair autophagic flux and lysosomal function, in part by activating mTOR and disrupting transcription factor EB (TFEB)–dependent lysosomal biogenesis [393,394]. In preclinical models, pharmacological or nutraceutical enhancement of autophagy—including modulation of mTOR/AMPK signalling (e.g., rapamycin [395] or spermidine [396]), polyphenols (resveratrol combined with vitamin E) [397], or emerging autophagy enhancers such as AUTophagy Enhancer-67 (AUTEN-67) [398]—has been shown in preclinical models to attenuate carbonyl stress, glycoxidation, and AGE-related proteotoxicity. However, clinical evidence remains limited. In addition, the use of synthetic agents that enhance antioxidant enzyme activity, such as SOD, represents a potential therapeutic option. Manganoporphyrins, EUK-134 and tempol, which mimic the action of SOD by counteracting free O_2_^•−^, are being studied [399]. Manganoporphyrins catalyse the conversion of O_2_^•−^ to H_2_O_2_ and oxygen, while EUK-134 is a salen–manganese complex that reduces oxidative stress [400]. Tempol, as a nitroxyl compound, detoxifies free radicals [399,400].

From a translational perspective, interventions aimed at restoring mitochondrial homeostasis may represent a complementary strategy to indirectly attenuate AGE–RAGE-driven neuroinflammation. Modulation of mitochondrial quality control, particularly activation of PINK1/PRKN-dependent mitophagy (e.g., through urolithin A or metformin), promotes the removal of damaged mitochondria, limits ROS production, and has been associated with reduced NLRP3 inflammasome activation and neuroinflammation, as well as improved cognitive function in animal models of AD [401,402]. In parallel, the use of mitochondria-targeted antioxidants (such as MitoQ) enables direct scavenging of mitochondrial ROS, stabilisation of mitochondrial membrane potential (ΔΨm), and preservation of ATP production, thereby reducing oxidative stress and partially alleviating AGE–RAGE axis activation and microglial reactivity [403,404]. Moreover, strategies that limit the release and activity of mtDAMPs, including mtDNA- or mitochondria-laden extracellular vesicles, may attenuate activation of innate immune and danger-sensing receptors, thereby reducing the chronic neuroinflammation observed in AD and PD [404]. Collectively, mitochondria-centred therapeutic approaches can help disrupt the inflammatory loop of AGE–RAGE-mitochondria that perpetuates itself and represent a promising direction for further translational investigation.

### 7.2 AGEs as potential biomarkers of neuropsychiatric disorders

Multiple AGE-related biomarkers are under investigation as tools for neurodegenerative disease assessment, including PEN in cerebrospinal fluid, circulating CML and CEL, and SAF measurements that capture tissue AGEs accumulation [264,[405], [406], [407]]. Importantly, higher concentrations of AGEs correlate with worsening cognitive function and the emergence of neurodegenerative pathology [408,409].

CML is one of the most common AGEs, formed mainly via oxidative degradation of Nε-fructosyl-lysine (FL) and lipid peroxidation–related pathways [410,411]. In the brain, CML primarily accumulates in neurons with neurofibrillary tangles and in amyloid plaques, where it co-occurs with tau protein and activates RAGE [410,411]. Immunohistochemical analyses have revealed a preferential accumulation of CML deposits in the brains of AD patients in the presence of type 2 diabetes. Comparative analyses of CML immunoreactivity revealed a stepwise gradient, with the highest signal intensity in individuals with concomitant AD and type 2 diabetes, followed by AD alone, then type 2 diabetes without dementia, and the lowest levels in physiological ageing [410,411]. In biological fluids such as cerebrospinal fluid, blood, and circulating extracellular vesicles (EVs), CML concentrations were elevated in patients with early or moderate AD, with parallel changes noted in type 2 diabetes serum, especially those with mild cognitive impairment. Importantly, CML levels showed a negative correlation with global cognitive performance, as assessed by the Mini-Mental State Examination (MMSE) [351,412,413]. Early elevation of CML levels is consistent with potential diagnostic and prognostic relevance in AD progression [412]. The diagnostic utility of CML can only be assessed once reference values for this biomarker have been established and the methods for measuring it have been validated in larger populations. CML can be detected in serum samples, brain tissue, and EVs using techniques such as enzyme-linked immunosorbent assay (ELISA), liquid chromatography–tandem mass spectrometry (LC–MS/MS), and immunohistochemistry (IHC) [412,414].

CEL is one of the AGEs formed from MGO and Lys residues. In the brain, it accumulates in Aβ plaques, particularly at Lys-16 and Lys-28, and in neurofibrillary tangles with tau protein, via RAGE–NF-κB activation and consequent oxidative and inflammatory stress [415]. Elevated levels of both CEL and immunoglobulin M (IgM) autoantibodies against CEL-modified apolipoprotein A1 (ApoA1) are found in the cerebrospinal fluid, plasma and EVs of patients with early and moderate AD [412,416,417]. Antibody levels are most prominent in early AD, indicating potential relevance for early-stage assessment [416]. CEL and anti-CEL antibodies are detected by LC–MS/MS, Western blot, and ELISA [416]. Although CML, CEL, and sRAGE are biologically plausible and potentially informative biomarkers, their current clinical validity remains limited. Available studies do not yet provide sufficiently consistent data on diagnostic sensitivity, specificity, or reproducible decision thresholds, and cross-study comparison is complicated by differences in sample source, assay methodology, patient selection, and metabolic comorbidity. In addition, incomplete preanalytical and analytical standardisation currently limits their routine clinical applicability, despite their mechanistic relevance [418,419].

Klimiuk et al. studied 50 patients with dementia, including 25 individuals with mild to moderate cognitive impairment (MMSE score of 11–23) and 25 individuals with severe dementia (MMSE score of 0–10), as well as 50 individuals from a control group [420]. The fluorescence intensity of AGEs in unstimulated and stimulated saliva was shown to increase correspondingly to the MMSE score decrease [421]. It was demonstrated that the level of AGEs in unstimulated saliva enabled the differentiation between mild/moderate and severe dementia with an area under the curve of 0.86 (sensitivity ≈ 80%, specificity ≈ 84%), while in stimulated saliva, it was 0.88 (sensitivity and specificity approximately 76%) [420]. Independently, Choromanska et al. studied 80 patients with moderate dementia and 80 age-matched healthy controls [421]. It was found that AGEs fluorescence in unstimulated saliva alone enabled effective differentiation between patients and healthy individuals, achieving an area under the curve of 0.85, with a sensitivity of 75.7% and a specificity of 75.9% [421]. Robust validation of salivary glycation markers will require analyses in larger, more diverse cohorts, with particular emphasis on defining assay-specific reference ranges and clarifying their diagnostic utility in neuropsychiatric disorders [422]. Indeed, clinical standardization of salivary AGEs measurements requires restriction to a well-characterised panel of analytes (e.g., CML, CEL, PEN) quantified using validated LC–MS/MS methods with stable isotope–labeled internal standards [423,424]. Immunochemical and fluorescence-based assays, although practical, exhibit limited analytical specificity and require calibration against LC–MS/MS reference measurements [424,425]. The lack of harmonized preanalytical protocols and standardized reporting remains a major translational barrier; therefore, until such standardization is achieved, salivary AGEs should be considered exploratory rather than routine clinical biomarkers [[423], [424], [425]]. Taken together, currently available AGE-related biomarkers should be regarded as promising but still exploratory translational candidates rather than routine clinical tools.

## 8 Future prospects

Current mechanistic frameworks increasingly incorporate carbonyl stress and AGE-related processes as contributors to neuropsychiatric disease vulnerability and progression within the CNS [426]. In patients with schizophrenia, PEN accumulation has been demonstrated in the frontal and parietal cortex, primary visual cortex, cerebellum, and basal ganglia, suggesting the existence of areas particularly sensitive to carbonyl stress in specific subpopulations of neurons [427]. It appears necessary to develop spatial mapping of the accumulation of individual types of AGEs in the brain, using high-resolution imaging techniques (*in vivo*), molecular analysis, and histology (post-mortem). Positron emission tomography (PET) has significant theoretical potential for *in vivo* assessment of AGE-related pathology in the human brain, primarily via imaging of RAGE as a surrogate marker of AGEs accumulation [428,429]. However, currently available first-generation RAGE PET tracers (such as [^18^F]RAGER and [^18^F]InRAGER) have limited specificity and insufficient clinical validation [428,429]. The development of second-generation tracers with improved selectivity, brain penetration, and pharmacokinetics, together with human imaging studies, is required before PET-based AGE/RAGE imaging can be considered a reliable biomarker [[428], [429], [430]]. This approach has the potential to clarify the links between region-specific carbonyl stress and neuropathological alterations, and to inform classification frameworks that account for underlying molecular heterogeneity in neuropsychiatric disorders. Existing preclinical data from animal models suggest that individual neuronal populations differ in their sensitivity to the accumulation of AGEs [47,427]. There is a lack of translational research involving human populations that takes into account species, environmental, and genetic differences. Similarly, sex-stratified evidence for differential AGEs accumulation or RAGE signalling in neuropsychiatric disorders remains limited, because most studies adjust for sex but rarely report sex-specific estimates or formally test sex-by-AGE/RAGE interactions [431,432]. From an epidemiological perspective, data indicating a dynamic increase in the number of dementia cases in East Asia are particularly significant [433]. One meta-analysis found significantly reduced levels of sRAGE in Asian populations, which may contribute to greater susceptibility to developing dementia [431]. Research on the AGE–RAGE axis across diverse populations may help define a more reliable panel of early biomarkers for neurodegeneration risk. The next step should be to find and test non-invasive methods for measuring AGEs, such as SAF (or its modifications), in clinical conditions, thereby enabling population screening [4]. Future biomarker development will likely require integrated multi-marker panels combining specific AGEs/AGE–RAGE-related measures with complementary signatures of oxidative stress, mitochondrial dysfunction, chronic inflammation, and other molecular classes (e.g., circulating proteins and DNA/RNA), in order to capture the complexity of metabolic–inflammatory networks more effectively than single-analyte approaches [434,435]. From a diagnostic perspective, future progress will require standardised analytical protocols, clinically meaningful cut-off validation, and prospective studies in well-characterised patient cohorts [436,437]. Potentially, the results of this screening could facilitate the identification of early phenotypes of neuropsychiatric disorders, thus improving therapeutic outcomes. The use of modern artificial intelligence-based computational methods—from graph algorithms and multiomics data analysis to generative models supporting drug design—may substantially advance the diagnosis and treatment of neuropsychiatric disorders associated with glycoxidation [438]. Expanding these areas of research would open up opportunities for developing targeted therapies based on the AGE–RAGE axis. Particularly promising future directions include multi-omics approaches linked to deep clinical phenotyping, advanced biomarker discovery pipelines, and mitochondria-targeted therapeutic strategies [404,439]. Given the central role of mitochondrial dysfunction in the self-amplifying AGE–RAGE–ROS loop described in this review, mitochondria-targeted interventions may be particularly relevant as future mechanism-based therapeutic strategies, including mitochondria-targeted antioxidants, modulation of mitochondrial biogenesis and dynamics, and enhancement of mitophagy aimed at interrupting the cascade of oxidative stress, inflammation, and neurodegeneration [401,440]. By combining multi-omics data with deep clinical phenotyping, artificial intelligence can reconstruct AGE–RAGE-related molecular networks and highlight the most promising, druggable nodes. Integrating these models with causal approaches (e.g., drug-target Mendelian randomisation) [441] helps separate true disease drivers from correlates, accelerating the prioritisation of AGE-linked targets for validation and drug repurposing/design [[441], [442], [443]].

## 9 Conclusions

Glycation- and glycoxidation-related processes are increasingly recognised as integral components of the molecular mechanisms underlying neurodegenerative and neuropsychiatric disorders. AGEs and associated oxidative stress are important contributors to cellular damage, especially in the nervous system [15,444]. High concentrations of AGEs/AOPPs induce carbonyl stress and inflammation through interactions with RAGE, which activates signalling pathways such as MAPK and PI3K/AKT, and subsequently NF-κB [445]. Acting as a multiligand pattern-recognition receptor, RAGE participates in inflammatory and redox-related signalling cascades that can reinforce carbonyl stress and promote further AGEs accumulation, thereby establishing a self-amplifying pathogenic loop [17,18,310]. In AD, the RAGE receptor activates Aβ storage, which accelerates neuronal degeneration [182,406]. Similarly, in PD, the AGE–RAGE interaction promotes αSyn deposition, which ultimately fosters neurodegeneration [1,174,446,447].

Currently, there are no neuropsychiatric drugs with well-documented direct antiglycation effects *in vivo*. Most available data instead describe indirect effects, primarily via redox modulation and altered AGE–RAGE-related signalling; combination with standard neuropsychiatric pharmacotherapy lacks clinical validation and remains experimental [17,448]. Among anti-dementia drugs, rivastigmine [323,324], galantamine [318,319], and memantine [317] show the most significant antiglycation potential. Among APDs, protective effects have been reported for risperidone [336], paliperidone [[332], [333], [334], [335]], and olanzapine [325,326], while clozapine [[327], [328], [329], [330], [331]] and haloperidol [331,337,338] may intensify glycation. Among antidepressants, antiglycation effects are attributed to paroxetine [346] and, indirectly, to escitalopram [348] and vortioxetine [349]. However, an important gap remains, as direct AGEs quantification is unavailable for many compounds and is infrequently assessed in clinical studies. This limitation hampers the translational interpretation of antiglycation effects reported for existing drugs. AGEs, especially CML [410,411] and CEL [416], show potential as prognostic and diagnostic biomarkers in neurodegenerative diseases but require further clinical validation.

Collectively, current evidence supports the involvement of glycation and glycoxidation mechanisms in molecular pathways relevant to the progression of neurodegenerative and neuropsychiatric diseases. Targeting elements of the AGE–RAGE axis and related redox processes may therefore represent a promising direction for complementary therapeutic strategies, pending further mechanistic and clinical validation [449]. Lifestyle interventions, including dietary AGEs restriction and regular physical activity, may indirectly attenuate carbonyl stress and protect brain function by reducing oxidative stress, neuroinflammation, and metabolic dysfunction, as supported by translational and experimental studies [426]. In animal models, high-AGE and high-fat diets exacerbate cognitive deficits and molecular brain pathology, whereas voluntary exercise reverses these changes and modulates AGE–RAGE signalling [450,451]. However, direct evidence demonstrating reductions in brain AGEs levels following lifestyle interventions in humans is currently lacking. Epidemiological data are limited to indirect systemic, metabolic, and cognitive correlates [452]. This may contribute to slowing disease progression; however, direct clinical evidence supporting such effects remains limited [3,17,174,292].

The priority areas for future research are long-term human studies aimed at clarifying the temporality/causality of carbonyl stress and AGE–RAGE activation in the early stages of the disease relative to its progression, as well as analytical standardization of glycation biomarkers (preferably using LC–MS/MS) [60,453]. In parallel, translational validation of *in vivo* approaches is needed—in particular, improved RAGE PET tracers and rigorously designed intervention studies targeting carbonyl stress/AGE–RAGE signalling — to determine whether modulating this axis pharmacologically/through lifestyle modifications can change clinically meaningful outcomes, not just surrogate markers [426,454]. From a translational perspective, progress in this field will require clearer separation of direct antiglycation activity from broader cytoprotective effects, together with robust *in vivo* validation and clinically relevant biomarker-based studies [455,456].

Summarising, future progress will likely depend on integrating multi-omics-based target discovery, advanced biomarker strategies, and mitochondria-focused therapeutic approaches into translational study design, through the combination of comprehensive genomic, transcriptomic, proteomic, and metabolomic profiling with robust computational integration and machine-learning methods to identify druggable mitochondrial pathways and clinically actionable signatures, validate them in relevant experimental models, and iteratively refine patient stratification, prognosis, and treatment-response prediction in well-designed clinical cohorts [[457], [458], [459], [460]].

## Declaration of generative AI and AI-assisted technologies in the writing process

During the preparation of this work the author(s) used Microsoft Copilot solely to improve language and readability only. After using this tool/service, the author(s) reviewed and edited the content as needed and take full responsibility for the content of the publication.

## Funding

This research was funded by the Medical University of Bialystok, Poland (grant number B.SUB.25.250). The APC was funded by the Medical University of Bialystok.

## CRediT authorship contribution statement

**Wiktor Orlof:** Conceptualization, Data curation, Investigation, Methodology, Validation, Visualization, Writing – original draft. **Mateusz Maciejczyk:** Conceptualization, Investigation, Supervision, Writing – original draft, Writing – review & editing.

## Declaration of competing interest

The authors declare that they have no known competing financial interests or personal relationships that could have appeared to influence the work reported in this paper.The author is an Editorial Board Member/Editor-in-Chief/Associate Editor/Guest Editor for this journal and was not involved in the editorial review or the decision to publish this article.

## Data Availability

No data was used for the research described in the article.

## Abbreviations

**3-DG**, 3-deoxyglucosone; **3 PG**, glycerol-3-phosphate; **4-HNE**, 4-hydroxynonenal; **5-HT**, serotonin; **5-HT2**, 5-hydroxytryptamine receptor 2; **8-oxoG**, 8-oxoguanine; **Aβ**, amyloid beta; **ACh**, acetylcholine; **AChEIs**, acetylcholinesterase inhibitors; **AD**, Alzheimer's disease; **ADTIQ**, 1-acetyl-6,7-dihydroxy-1,2,3,4-tetrahydroisoquinoline; **AEDs**, antiepileptic drugs; **AGEs**, advanced glycation end products; **ALEs**, advanced lipoxidation end products; **ALDH**, aldehyde dehydrogenase; **AOPPs**, advanced oxidation protein products; **ApoA1**, apolipoprotein A1; **apoE**, apolipoprotein E; **APP**, amyloid precursor protein; **APDs**, antipsychotic drugs/neuroleptics; **APs**, Amadori products; **AR**, aldose reductase; **ARG**, argpyrimidine; **Arg**, arginine; **ATP**, adenosine 5′-triphosphate; **BACE1**, beta-site APP cleaving enzyme 1; **Bax**, Bcl-2-associated X protein; **BBB**, blood–brain barrier; **Bcl-2**, B-cell lymphoma 2; **BDNF**, brain-derived neurotrophic factor; **BH_4_**, tetrahydrobiopterin; **BLT1**, leukotriene B4 receptor 1; **BMI**, body mass index; **BSA**, bovine serum albumin; **CAT**, catalase; **Casp3**, caspase-3; **CEL**, Nε-(carboxyethyl)lysine; **CMA**, Nω-(carboxymethyl)arginine; **CML**, Nε-(carboxymethyl)lysine; **CNS**, central nervous system; **CoQ10**, coenzyme Q10; **COMTIs**, catechol-*O*-methyltransferase inhibitors; **Cys**, cysteine; **C1q**, complement component 1q; **C3a**, complement component 3a; **DA**, dopamine; **DAs**, dopamine agonists; **DIAPH1**, diaphanous-related formin 1; **DJ-1**, Parkinson disease protein 7; **DNA**, deoxyribonucleic acid; **Drp1**, dynamin-related protein 1; **ECM**, extracellular matrix; **EGF**, epidermal growth factor; **ELISA**, enzyme-linked immunosorbent assay; **eNOS**, endothelial nitric oxide synthase; **ER**, endoplasmic reticulum; **ERK1/2**, extracellular signal-regulated kinase 1/2; **esRAGE**, endogenous secretory receptor for advanced glycation end products; **ETC**, electron transport chain; **EVs**, extracellular vesicles; **F3P**, fructose-3-phosphate; **FGAs**, first-generation antipsychotics; **FL**, Nε-fructosyl-lysine; **FPRs**, formyl peptide receptors; **G6P**, glucose-6-phosphate; **GLO1/2**, glyoxalase 1 and 2; **GLUT4**, glucose transporter type 4; **GLYR**, glyoxylate reductase; **GO**, glyoxal; **GPx**, glutathione peroxidase; **GPx4**, glutathione peroxidase 4; **GRP78**, glucose-regulated protein 78; **GSH**, glutathione; **HbA1c**, glycated haemoglobin; **HMGB1**, high-mobility group box 1; **HSP70**, heat shock protein 70; **H_2_O_2_**, hydrogen peroxide; **ICAM-1**, intercellular adhesion molecule 1; **IDO1**, indoleamine 2,3-dioxygenase 1; **IFN-α**, interferon alpha; **IFN-γ**, interferon gamma; **IgM**, immunoglobulin M; **IHC**, immunohistochemistry; **IKK**, inhibitor of κB kinase; **IκBα**, inhibitor of κB alpha; **IL-1**, interleukin 1; **IL-1β**, interleukin 1 beta; **IL-2**, interleukin 2; **IL-6**, interleukin 6; **IL-8**, interleukin 8; **IL-18**, interleukin 18; **iNOS**, inducible nitric oxide synthase; **JNK**, c-Jun N-terminal kinases; **KAT I/II**, kynurenine aminotransferase I and II; **KEAP1**, Kelch-like ECH-associated protein 1; **LC–MS/MS**, liquid chromatography–tandem mass spectrometry; **LRRK2**, leucine-rich repeat kinase 2; **LPS**, lipopolysaccharides; **LSD**, lysergic acid diethylamide; **LTP**, long-term potentiation; **Lys**, lysine; **M17**, M17 neuroblastoma cell line; **MAO-B**, monoamine oxidase B; **MAOIs**, monoamine oxidase inhibitors; **MAPK**, mitogen-activated protein kinase; **MAPKK**, MAP kinase kinase; **MAPKKK**, MAP kinase kinase kinase; **MCP-1**, monocyte chemoattractant protein 1; **MDA**, malondialdehyde; **MGO**, methylglyoxal; **MHC-II**, major histocompatibility complex class II; **MMSE**, Mini-Mental State Examination; **MPO**, myeloperoxidase; **MPP^+^**, 1-methyl-4-phenylpyridinium; **MPTP**, 1-methyl-4-phenyl-1,2,3,6-tetrahydropyridine; **mtDAMPs**, mitochondrial damage-associated molecular patterns; **mtDNA**, mitochondrial DNA; **mtROS**, mitochondrial reactive oxygen species; **mTOR**, mechanistic target of rapamycin; **MyD88**, myeloid differentiation primary response 88; **NA**, noradrenaline; **NAC**, N-acetylcysteine; **NAD^+^**, oxidised nicotinamide adenine dinucleotide; **NADPH**, reduced nicotinamide adenine dinucleotide phosphate; **NF-κB**, nuclear factor kappa-light-chain-enhancer of activated B cells; **NLRP3**, NOD-like receptor family pyrin domain containing 3; **NMDA**, N-methyl-d-aspartate; **NO**, nitric oxide; **NOS1/2**, nitric oxide synthase 1 and 2; **NOX1/2/4**, NADPH oxidase isoforms 1, 2, and 4; **Nrf2**, nuclear factor erythroid 2-related factor 2; **ONOO^−^**, peroxynitrite; **O_2_•^-^**, superoxide anion radical; **PAMPs**, pathogen-associated molecular patterns; **PC12**, pheochromocytoma cell line; **PCOs**, protein carbonyls; **PD**, Parkinson's disease; **PEN**, pentosidine; **PET**, positron emission tomography; **PHFs**, paired helical filaments; **PI3K/AKT**, phosphatidylinositol 3-kinase/protein kinase B; **PINK1**, PTEN-induced putative kinase 1; **PKC**, protein kinase C; **PRKN**, parkin; **PRRs**, pattern recognition receptors; **PS1**, presenilin 1; **PSM**, proteasome peptidase; **PUFAs**, polyunsaturated fatty acids; **Rac1**, Ras-related C3 botulinum toxin substrate 1; **RAGE**, receptor for advanced glycation end products; **RCS**, reactive carbonyl species; **RNA**, ribonucleic acid; **RNS**, reactive nitrogen species; **ROS**, reactive oxygen species; **SAF**, skin autofluorescence; **SAPK**, stress-activated protein kinase; **SDH**, sorbitol dehydrogenase; **SGAs**, second-generation antipsychotics; **SH-SY5Y**, human neuroblastoma cell line; **SIRT1**, sirtuin 1; **SNRI**, serotonin–noradrenaline reuptake inhibitor; **SOD1/2**, superoxide dismutase 1 and 2; **SSRI**, selective serotonin reuptake inhibitor; **sRAGE**, soluble receptor for advanced glycation end products; **TBARS**, thiobarbituric acid reactive substances; **TCAs**, tricyclic antidepressants; **TFEB**, transcription factor EB; **TIRAM**, adaptor protein related to TIR domain (TIRAP); **TLRs**, Toll-like receptors; **TNF-α**, tumour necrosis factor alpha; **TPH1/2**, tryptophan hydroxylase 1 and 2; **TrkB**, tropomyosin receptor kinase B; **Trp**, tryptophan; **TXNIP**, thioredoxin-interacting protein; **UCHL1**, ubiquitin C-terminal hydrolase L1; **UPS**, ubiquitin–proteasome system; **VCAM-1**, vascular cell adhesion molecule 1; **VES**, vesperlysine; **WHO**, World Health Organization; **αSyn**, alpha-synuclein; **•OH**, hydroxyl radical.
