# Supplementary material for: Protein glycoxidation in neuropsychiatric disorders—from basic research to clinical practice
Source: Redox Biol. 2026 Apr 27;94:104190. doi: 10.1016/j.redox.2026.104190 (PMC13181251; doi:10.1016/j.redox.2026.104190)
Supplement: Multimedia component 1 [file mmc1.docx]

**Supplementary Table S1. Neurological and psychiatric drugs evaluated for their antiglycation activity (inhibition of the formation and signalling of advanced glycation end products (AGEs))—review of *in vitro* / *in vivo* evidence, proposed molecular mechanisms, and current state of research.**

| **Drug** | **Has a direct antiglycation effect been demonstrated?** | **A brief description of the mechanism of action** | **Citation** |
| --- | --- | --- | --- |
| **Antiparkinsonian drug** | | | |
| **Amantadine** increases extracellular dopamine (DA) concentration by enhancing its release in the striatum and by blocking reuptake by presynaptic neurons | - No / proglycation effect (*in vitro*) | - Amantadine does not significantly inhibit protein glycation or glycoxidation in a bovine serum albumin (BSA) model. Its effect in this regard is minimal or indirect, and under certain conditions, a proglycoxidation effect has even been observed | [336] |
| **Apomorphine**, a potent dopamine agonist (DAs) | - Yes (*in vitro*) | - The inhibition of the BSA reaction with glucose results from the catechol structure of apomorphine, which enables the chelation of Fe²⁺ ions and enhanced control of intracellular reactive oxygen species (ROS) levels. This has the effect of reducing the formation of protein carbonyls (PCOs) and inhibiting glycoxidation | [333] |
| **Bromocriptine** is a semi-synthetic derivative of ergotamine (ergot alkaloid), a DAs | - Yes, but indirect rationale (*in vivo*) | - Reduces carbonyl stress (in rat models of streptozotocin-induced diabetes and human type 2 diabetes), as reflected in a reduction in glycated haemoglobin A1c (HbA1c) and thus a decrease in AGEs. It also shows antioxidant properties, but has not been evaluated in classic Maillard reaction assays | [334,335] |
| **Drugs used to treat dementia, including Alzheimer’s disease (AD)** | | | |
| **Memantine** (N-methyl-D-aspartate (NMDA) receptor antagonist) | - Yes (*in vitro*) | - Inhibits AGE-induced degradation of the extracellular matrix (ECM) and suppresses activation of the Janus kinase 2/signal transducer and activator of transcription 1 (JAK2/STAT1) pathway in human chondrosarcoma cell line (SW1353),  - The authors point to its carbonyl-scavenging potential | [344] |
| **Galantamine** (acetylcholinesterase inhibitor (AChEIs) | - Yes (*in vivo*) | - Used in Wistar rats in a model of colitis induced by 2,4,6-trinitrobenzenesulfonic acid, it acted as an agonist of the alpha7 nicotinic acetylcholine receptor (α7 nAChR), leading to inhibition of the (JAK2/STAT3) and nuclear factor κB (NF-κB) pathways, as well as high-mobility group box 1/receptor for advanced glycation end products (HMGB1/RAGE), and increased interleukin 10 (IL-10) levels  - Administered to transgenic mice (APP23) in a hypoperfusion AD model, it reduces AGEs levels, including Nε-(carboxymethyl)lysine (CML) and Nω-(carboxymethyl)arginine (CMA), inhibits matrix metalloproteinase 9 (MMP9) activity, and promotes neuroprotection through activation of the brain-derived neurotrophic factor/tropomyosin receptor kinase B (BDNF/TrkB) pathway | [345,346] |
| **Donepezil** (AChEIs) | - Yes (*in vivo*)—partially confirmed, but a proglycation effect is also observed | - Lowers AGEs levels in the brains of C57BL/6J mice fed a high-fat diet and Sprague Dawley rats with AD induced by amyloid beta (Aβ) and Wistar rats with streptozotocin-induced diabetes, and also exhibits antioxidant properties (increases reduced glutathione (GSH) levels and reduces malondialdehyde (MDA)) and anti-inflammatory properties (reduces tumour necrosis factor alpha (TNF-α) and interleukin-6 (IL-6)), reduces Aβ levels and shows neuroprotective properties,  - Donepezil in a streptozotocin-induced diabetic rat model increased HbA1c and Aβ (in blood), indicating increased glycation in hyperglycaemia | [347–349] |
| **Rivastigmine** (AChEIs) | - Yes (*in vivo*) | - Clinical trials in patients with AD have shown that rivastigmine reduces AGEs, activates the sirtuin 1 (SIRT1) pathway, increases reduced GSH levels, and decreases ROS production. It also inhibits the translocation of NF-κB and reduces the expression of pro-inflammatory cytokines such as TNF-α and IL-6. In addition, it affects the modulation of the mechanistic target of rapamycin (mTOR) pathway signalling | [350,351] |
| **Drugs used in the treatment of psychotic disorders (schizophrenia)** | | | |
| **Olanzapine** (second-generation antipsychotic, SGAs) | - Yes (*in vivo* and *in vitro*) | - Decreased levels of the soluble receptor for AGEs (sRAGE) in the blood of patients with first-episode schizophrenia may indicate indirect suppression of AGE–RAGE signalling,  - Inhibited PCOs formation in a rat model of chronic social isolation stress (resulting in depression) | [352,353] |
| **Clozapine** (SGAs) | - No (*in vitro* and *in vivo*), proglycation effect | - Increases PCOs levels, enhances lipid peroxidation and ROS formation as indicated by proglycation and pro-oxidant effects in both (Wistar) rats with oxidative damage in the brain and heart and in the (PC12) cell lines used to study neurodegeneration | [354–358] |
| **Paliperidone** (SGAs), an active metabolite of risperidone | - Yes (*in vitro* and *in vivo*) | - Exhibits anti-AGE activity confirmed *in silico* and *in vitro* studies (MCF-7 human breast cancer cell line); characterised by strong binding to RAGE and reduced expression of RAGE and HMGB1,  - Demonstrates potential neuroprotective benefits in Sprague Dawley rat brains and PD cell lines (SH-SY5Y) due to its ability to inhibit the increase in oxidative stress markers | [359–362] |
| **Risperidone** (SGAs) (oral and long-acting injectable (LAI)) | - Yes (*in vivo*) | - In C57BL/6 mice exposed to 1,2-diacetylbenzene (a strong inducer of AGEs), administration of risperidone reduced AGEs (CML) levels in the hippocampus and inhibited the activation of the RAGE–NF-κB pathway, alleviating oxidative stress | [363] |
| **Haloperidol** (first-generation antipsychotic, FGAs) | - No (*in vivo*), proglycation effect | - In a rat model of haloperidol treatment, increased PCOs levels in the hippocampus and increased concentrations of thiobarbituric acid reactive substances (TBARS) were observed, indicating a proglycation effect,  - In a human (schizophrenia) model, plasma PCOs levels were higher in patients treated with haloperidol | [358,364,365] |
| **Quetiapine** (SGAs) | - No (*in vivo*)—partially confirmed,  - The results are inconclusive; antioxidant effects have also been observed | - The observed increase in HbA1c even at low doses indicates a factor promoting glycation (in a human model with mental health disorders treated with quetiapine),  - Studies suggest that long-term use of quetiapine may reduce oxidative stress and Aβ accumulation (in models of amyloid precursor protein/presenilin-1 double transgenic mice); Sprague Dawley rats (chronic mild stress model), and in cell lines used to study neurodegeneration (PC12 cells, e.g. with Aβ) | [366–370] |
| **Drugs used in the treatment of depressive disorders** | | | |
| **Agomelatine**, a noradrenaline–dopamine disinhibitor | - No (*in vitro*)—shows a pro-AGE effect | - In the BSA test with the addition of glucose, fructose, glyoxal (GO), methylglyoxal (MGO), it did not show the ability to reduce AGEs fluorescence or PCOs levels; under certain conditions, an intensification of glycation was even observed compared to the control. The lack of a carbonyl-scavenging effect may be explained by the weak binding of agomelatine to BSA | [371] |
| **Trazodone** shows a mixed mechanism of action and affects serotonergic conduction | - No (*in vitro*)—appears to enhance glycation | - Studies and a systematic review showed that it enhanced the formation of AGEs and advanced oxidation protein products (AOPPs) in a model of BSA with sugars or aldehydes (GO, MGO). In comparison, standard antiglycation compounds, such as aminoguanidine, showed significantly greater efficacy | [372] |
| **Paroxetine** (selective serotonin reuptake inhibitor, SSRI) | - Yes (*in vitro* and *in vivo*)—partially confirmed | - *In vitro* hyperglycaemic models included bEnd.3 (mouse) and EA.hy926 (human) cell lines for the assessment of endothelial damage and Sprague Dawley rats with streptozotocin-induced diabetes in an *in vivo* model. The tested drug reduces oxidative stress by limiting the production of mitochondrial ROS, protein oxidation, and deoxyribonucleic acid (DNA) damage in endothelial cells, and improves vascular function in rats; it may indirectly inhibit the AGE–RAGE cascade by reducing reactive carbonyl species (RCS) and ROS (as postulated by the authors) | [373] |
| **Fluoxetine** (SSRI) | - Yes (*in vivo*)—partially confirmed | - In a chronic mild stress-induced model in Sprague Dawley rats, it restored S100B protein and RAGE expression in the hippocampus, but AGEs levels were not determined, and its anti-AGE effect remains hypothetical, as postulated by the authors | [374] |
| **Escitalopram** (SSRI) | - Yes (*in vivo*)—partially confirmed | - In a streptozotocin-induced rat diabetes model, it improved the glycaemic profile and reduced RAGE, NF-κB, and pro-inflammatory cytokines; AGEs were not measured, but lower RAGE means a weaker response to existing AGEs | [375] |
| **Vortioxetine** (multimodal serotonin modulator) | - Yes (*in vivo*)—partially confirmed | - In a rotenone-induced neurodegeneration model in Sprague Dawley rats, it reduced the expression of S100B protein, RAGE, and NF-κB in intestinal glial cells; this suggests attenuation of AGE–RAGE-related signalling | [376] |
| **Table abbreviations**: **AD**, Alzheimer’s disease; **AGEs**, advanced glycation end products; **AOPPs**, advanced oxidation protein products; **Aβ**, amyloid beta; **BDNF**, brain-derived neurotrophic factor; **BSA**, bovine serum albumin; **CMA**, Nω-(carboxymethyl)arginine; **CML**, Nε-(carboxymethyl)lysine; **DAs**, dopamine agonists; **DA**, dopamine; **DNA**, deoxyribonucleic acid; **ECM**, extracellular matrix; **FGAs**, first-generation antipsychotic; **GO**, glyoxal; **GSH**, reduced glutathione; **HMGB1**, high mobility group box 1; **HbA1c**, glycated haemoglobin A1c; **AChEIs**, acetylcholinesterase inhibitor; **IL-6**, interleukin 6; **IL-10**, interleukin 10; **JAK2**, Janus kinase 2; **LAI**, long-acting injectable; **MDA**, malondialdehyde; **MGO**, methylglyoxal; **MMP9**, matrix metalloproteinase 9; **mTOR**, mechanistic target of rapamycin; **NF-κB**, nuclear factor κB; **NMDA**, N-methyl-D-aspartate; **NA**, noradrenaline; **PCOs**, protein carbonyl; **RAGE**, receptor for AGEs; **RCS**, reactive carbonyl species; **ROS**, reactive oxygen species; **S100B**, S100B protein; **SGAs**, second-generation antipsychotic; **SIRT1**, sirtuin 1; **sRAGE**, soluble RAGE; **SSRI**, selective serotonin reuptake inhibitor; **STAT1/3**, signal transducer and activator of transcription 1/3; **SW1353**, chondrosarcoma cell line SW1353; **TBARS**, thiobarbituric acid reactive substances; **TNF-α**, tumour necrosis factor α; **TrkB**, tropomyosin receptor kinase B; **5-HT**, 5-hydroxytryptamine (serotonin); **α7 nAChR**, alpha7 nicotinic acetylcholine receptor. | | | |
